# m6A-Regulator Expression Signatures Identify a Subset of Follicular Lymphoma Harboring an Exhausted Tumor Microenvironment

**DOI:** 10.3389/fimmu.2022.922471

**Published:** 2022-06-06

**Authors:** Tingting Zhang, Hengqi Liu, Fenghua Gao, Wenchen Gong, Yaoli Cui, Jin He, Lanfang Li, Lihua Qiu, Zhengzi Qian, Shiyong Zhou, Bin Meng, Xiubao Ren, Huilai Zhang, Xianhuo Wang

**Affiliations:** ^1^ Department of Lymphoma, Tianjin Medical University Cancer Institute and Hospital, National Clinical Research Center for Cancer, Tianjin, China; ^2^ Key Laboratory of Cancer Prevention and Therapy, Tianjin’s Clinical Research Center for Cancer, The Sino-US Center for Lymphoma and Leukemia Research, Tianjin, China; ^3^ Department of Pathology, Tianjin Medical University Cancer Institute and Hospital, Tianjin, China; ^4^ Department of Immunology/Biotherapy, Tianjin Medical University Cancer Institute and Hospital, Tianjin, China

**Keywords:** m6A, tumor microenvironment, immunotherapy, follicular lymphoma, exhaustion

## Abstract

The role of N6-methyladenosine (m6A) modification in tumor microenvironment has rarely been explored in follicular lymphoma (FL). To examine the role of m6A modification in biological behavior, especially the immune landscape of FL, we utilized the Gene Expression Omnibus database to determine the expression signatures of m6A-regulators by unsupervised clustering, and then condense into a risk score, which was validated in an external cohort from the Tianjin Medical University Cancer Institute and Hospital. Finally, 16 m6A-regulators in 351 FL patients were evaluated and two m6A clusters were identified, characterized by differences in prognosis and biological behaviors. The m6A score was further developed based on 20-genes to quantify the m6A-regulator expression signature in each patient with FL. The low m6A score was associated with inferior prognosis of patients, with a median survival time of 8.84 (95% confidence interval [CI]: 7.251-10.429) years, which was remarkably shorter than that of patients with high m6A scores (15.73 years, 95% CI: 11.729-19.731; p<0.0001). Genes like TNFRSF14, CREBBP, and CARD11 were shown to be more often mutated in the low m6A group. This group was enriched with immune/inflammatory response but along with the abundant infiltration of exhausted T cells and the upregulated PD-1 and PD-L1 expression. Finally, we verified the m6A score could predict the response to anti-PD-L1 antibodies in an immunotherapy cohort. To conclude, the m6A score recognizes a section of FL patients harboring an exhausted tumor microenvironment and may help guide more effective immunotherapy strategies for patients with FL.

## Introduction

Follicular lymphoma (FL), the most common type of indolent B cell non-Hodgkin lymphoma, is a highly heterogeneous disease with variable biological and clinical behaviors (1). Despite therapeutic advances during the past decades and median overall survival (OS) duration of more than 10 years, 20-30% of patients will experience early disease progression, and subsets of individuals suffering from FL are more prone to mortality from this disease (2, 3). Still, the basic molecular processes that lead to the pathological development of FL are not clearly understood. Hence, a deep understanding of FL oncogenesis and development is important to facilitate a more personalized approach to the management of FL.

Epigenetic dysregulation is crucial for the pathological development of FL (4, 5). Recently, N6-methyladenosine (m6A), the most common posttranscriptional modification of mRNA, has attracted great attention in the cancer research field. m6A modification influences a series of biological functions, including RNA stabilization, splicing, export, translation, and degradation (6–10). Accumulating evidence indicates that m6A modification is extensively involved in the development and progression of multiple non-hematological and hematological cancers (11). Research has revealed that m6A modification could increase the self-renewal ability of acute myeloid leukemia cells and contribute to the pathogenesis of acute myeloid leukemia (12–14). The function of m6A modification in lymphoma has also been reported. For example, WTAP, an m6A methyltransferase, was markedly upregulated in nasal-type natural killer/T-cell lymphoma, and WTAP-guided m6A methylation contributed to tumor cell proliferation and chemotherapy resistance (15). Cheng Y and colleagues found that the m6A methylation level was upregulated in diffuse large B-cell lymphoma (DLBCL) and that METTL3 promoted disease progression (16). However, the role of m6A modification in FL is largely unknown.

Extensive studies have suggested that m6A modification is important for both innate and adaptive immune modulation, and also to shape the tumor microenvironment (TME) (17, 18). In solid tumors, such as gastric cancer and clear cell renal carcinoma, m6A modification is closely connected to distinct immune subtypes and cell infiltration characteristics (19, 20). FL is characterized by the infiltration of a substantial number of T cells in TME, which has a significant impact on the FL biology and outcome (5). However, the role of m6A modification in the formation of TME diversity and complexity in FL, is poorly understood.

m6A modification, a reversible and dynamic process, is mainly regulated by three categories of proteins: “writers” (methyltransferases), “readers” (effector proteins), and “erasers” (demethylases). The measuring techniques of m6A modification level includes MeRIP-seq, miCLIP-seq, SCARLET, LC-MS/MS and so on (21). All those detection methods have their own limitations and their application in clinical practice remains challenge. Nevertheless, the m6A modification level is closely related to the expression level of writers, readers, and erasers. Hence, we evaluated the interaction role of m6A-regulators instead directly determining the m6A modification level for the convenience of practical application. In this study, we comprehensively evaluated the expression of 16 m6A-regulators in 351 FL patients from three cohorts, examined different m6A-regulator expression signatures and further identified a subset of patients prone to therapeutic effects of immune therapy.

## Methods

### Data Collection and Processing

The Gene Expression Omnibus (GEO) database was thoroughly searched for all eligible FL datasets. Then, three datasets, GSE16131 (N=184), GSE119214 (N=137), and GSE66166 (N=138), were enrolled. Gene expression data and related clinical details from the three datasets were downloaded from the GEO database (https://www.ncbi.nlm.nih.gov/geo/). Notably, the samples in both GSE119214 and GSE66166 were obtained from the BC Cancer Agency cohort. Hence, only 30 samples in GSE66166 were subjected to further analysis after removing the 108 duplicated samples in GSE119214. Finally, in this research, total 351 patients were recruited, of whom 317 had available survival data. Detailed clinical information of each dataset is provided in **Table S1**. The batch effect was removed using ComBat from the R package sva (version 3.40.0) (22).

Fresh frozen tumor biopsies from 77 FL patients were acquired from the Tianjin Medical University Cancer Institute and Hospital. After excluding patients with grade 3b, histologic transformed FL, FL with concurrent DLBCL and with incomplete follow-up data, 41 patients being provided with standard first-line therapy were recruited for the validation cohort. For these patients, sufficient amount of quality genomic RNA (n=41) and DNA (n=38) from tumor biopsies were extracted for high-throughput sequencing. The Tianjin Medical University Cancer Institute and Hospital’s Clinical Research Ethics Board provided the approval for this study. Informed written consents were acquired. The flow diagram of our study is briefed in **Figure S1**.

### Unsupervised Clustering of 16 m6A-Regulators

Total 16 m6A-regulators were isolated from the integrated GEO database, including eight writers (WTAP, RBM15, RBM15B, ZC3H13, METTL3, METTL14, KIAA1429, and CBLL1), seven readers (HNRNPC, HNRNPA2B1, YTHDC1, YTHDF2, IGF2BP1, ELAVL1, and LRPPRC) and one eraser (ALKBH5). The STRING database (http://www.db.org/) was utilized to analyse the interactive network of these m6A-regulators. Unsupervised clustering analysis was conducted using the ConsensusClusterPlus package (version 1.56.0) (23) based on the k-means algorithm to evaluate the distinct expression signatures of m6A-regulators. The Euclidean distance was utilized as the distance measure, and 1000 bootstrap replications were performed.

### Gene Set Annotation Enrichment Analysis

Gene set variation analysis (GSVA) was performed to identify the distinct biological processes between the expression signatures of m6A-regulators using GSVA R package (version 1.40.1) (24). The ‘c2. cp. kegg. v6.2. symbols’ gene set, obtained from Molecular Signatures Database, was used for GSVA. A statistical significance of adjusted p<0.05 was used in the analysis. Gene set enrichment analysis (GSEA) was performed using the Java desktop software (version 4.1.0) (25, 26). The significance threshold was established at |normalized enrichment score|>1, nominal p-value < 0.05.

### Identification of Differentially Expressed Genes (DEGs)

Patients were grouped into distinct m6A clusters based on m6A regulator expression to identify genes that correlated with m6A-regulator expression signature. DEGs between m6A clusters were determined using the limma package (version 3.48.3) (27). Genes with an adjusted p<0.01 were identified as differentially expressed.

### Generation of the m6A-Related Cluster and m6A Score

To better characterize the underlying biological differences between m6A clusters, we identified m6A-related clusters based on the DEGs identified from m6A clusters by unsupervised clustering analysis. On top of that, we developed a scoring model to quantify the m6A-regulator expression signature in each patient. Briefly, we utilized the random forest approach to remove redundant DEGs obtained in the previous step. The remaining genes’ predictive significance was assessed *via* Univariate Cox regression analysis. Genes with statistical significance of p<0.05 were subjected to further analysis. Then, principal component analysis (PCA) was conducted and both principal components 1 and 2 were plotted to quantify the m6A-regulator expression signature, termed the m6A score. The calculation formula was as follows:


m6A score=∑PC1i+∑PC2i

where i is the expression of DEGs with significant prognostic value between the m6A clusters.

### RNA Sequencing and Gene Expression Analysis

RNeasy Kit (Qiagen, Hilden, Germany) was utilized to isolate RNA. RNAs libraries were created *via* NEBNext^®^ UltraTM RNA Library Prep Kit and sequenced based on Illumina NovaSeq 6000 platform (San Diego, CA, USA) with 150 bp paired-end reads. Subsequently, reads were aligned to the Human Genome Reference Consortium build 37(GRCh37/hg19). Fragments per kilobase per million (FPKM) were utilized to standardize the expression values of each gene. Then, m6A score was calculated to measure the m6A-regulator expression signature in each patient.

### Whole Exome Sequencing and Identification of Somatic Single Nucleotide Variations and Indels

DNeasy Tissue and Blood Kit (Qiagen, Venlo, Netherlands) were utilized to isolate DNA. Libraries were created *via* Agilent SureSelect Human All Exon kit V6 (Agilent Technologies, CA, USA) and sequenced on an Illumina NovaSeq 6000 platform with 150 bp paired-end reads. Valid sequencing data were then aligned to the GRCh37/hg19 by BWA (v0.7.12) (28). Then, SAM tools (29), Picard (v1.87) and Genome Analysis Toolkit (GATK) (30) were used to sort BAM files and performing repeated marking, local realignment, and base quality recalibration. Single nucleotide variants (SNVs) were identified using the GATK Unified Genotyper and indels were determined using VarScan. ANNOVAR package (31) were used for annotation of all substitutions and indels. Recurrent mutated genes in FL reported in previous literature were selected and further analysed (5).

### Estimation of Infiltrating Immune Cells

The relative abundance of six immune cell subtypes was evaluated by TIMER (https://cistrome.shinyapps.io/timer/) (32). Immune Cell Abundance Identifier (ImmuCellAI) was further utilized to specifically evaluate the abundance of 18 comprehensive T-cell subpopulations (http://bioinfo.life.hust.edu.cn/web/ImmuCellAI/) (33).

### Prediction of the Response to Anti-PD-L1 Therapy

An immunotherapeutic cohort of patients with advanced urothelial cancer administered with atezolizumab, an anti-PD-L1 antibody (IMvigor210 cohort, N=354), was enrolled in this study to predict the response to immunotherapy (34). The expression data and corresponding clinical information were downloaded from http://research-pub.Gene.com/imvigor210corebiologies. The FPKM value was converted from the raw count value and m6A score was calculated. Then, receiver operating characteristic (ROC) curve analysis was conducted to compare the performance of m6A score with other predictive biomarkers in predicting the response of anti-PD-L1 therapy, such as PD-L1 expression on tumor cells, tumor immune dysfunction and exclusion (TIDE) (35), and tumor inflammation signature (TIS) (36). PD-L1 expression data was downloaded from https://research-pub.Gene.com/imvigor210corebiologies. After the expression data quantile normalized, TIDE score was calculated using the web application (HTTPS://tide.dfci.harvard.edu/) and TIS score was calculated as an average value of 18 signature gene expression after log10 transformed.

### Statistical Analysis

The Wilcoxon two-tailed test was utilized for comparing two groups. Correlations were evaluated by Spearman analysis. Comparison for the survival curves was dawn *via* the Kaplan-Meier log-rank test. p<0.05 was of statistical significance. The optimal cutoff point of the m6A score was calculated using the “survminer” package (version 0.4.9) for the survival analysis. ROC curves with the “pROC” package (version 1.18.0) and time-dependent ROC curves with “survivalROC” package (version 1.0.3) were used to compare the performance of the m6A score. All the analysis was performed *via* R (version 4.1.0).

## Results

### Landscape of m6A-Regulators in FL

A total of 16 m6A-regulators were ultimately identified in this study: eight writers, seven readers, and one eraser. **Figure S2A** showed the locations of the m6A-regulators on chromosomes. As m6A methylation is involved in the interaction of writers, erasers, and readers, we then analyzed the correlation of m6A-regulator expression. We found that these regulators had complex and interlaced correlations with each other (**Figures S2B, C**; **Table S2**), highlighting the need to identify potential expression signatures to elucidate the clinical significance of these m6A-regulators.

### m6A Clusters Mediated by 16 m6A-Regulators

**Figure 1:**
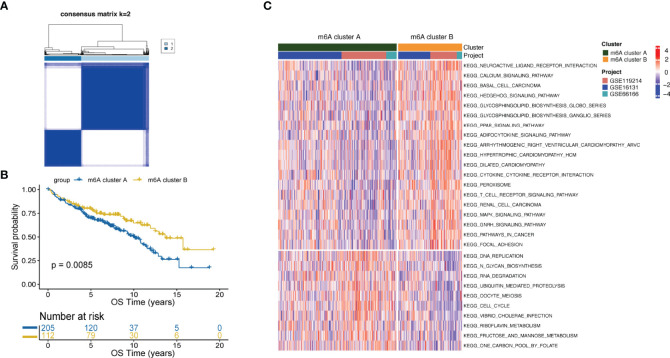
m6A clusters mediated by m6A-regulators and the biological features of each cluster. **(A)** Unsupervised clustering based on m6A-regulator expression and consensus matrices for k = 2. **(B)** Survival analysis between m6A clusters by the log-rank test. **(C)** GSVA showing the activation status of biological pathways in m6A cluster A vs. m6A cluster B. An adjusted p-value<0.05 was considered significantly significant. Biological pathways with adjusted p<0.05 and p<0.0001 were shown in the figure.

FL patients were classified based on the expression data of 16 m6A-regulators using unsupervised clustering. As per the similarities shown by 16 m6A-regulators expression, k-means clustering was carried out numerous times for different values of k (k = 2-10), and k = 2 was found as having the best clustering stability. (**Figures 1A**, **S3**). Ultimately, two different m6A-regulator expression signatures were identified, termed m6A clusters A and B. Prognostic analysis revealed that m6A cluster A was significantly associated with poorer outcomes as compared to m6A cluster B (p=0.0085; **Figure 1B**). To explore differences in biological behaviors between the two m6A clusters, we performed GSVA. Significantly differentially activated pathways (adjusted p<0.05) between the two groups were summarized in **Table S3**. Specifically, m6A cluster A showed enrichment in the DNA replication, RNA degradation and cell cycle. However, m6A cluster B had a significant enrichment in oncogenic pathways such as the MAPK signaling pathway and hedgehog signaling pathway (**Figure 1C**). Particularly, immune pathways, such as the cytokine-cytokine receptor interaction and T-cell receptor signaling, were activated in m6A cluster B (**Figure 1C**), suggesting that m6A modifications play certain roles in the TME of FL.

### Construction of the m6A-Related Cluster

**Figure 2:**
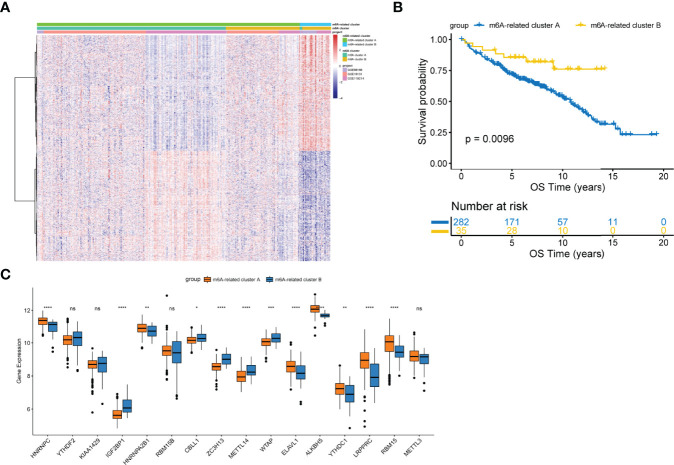
Construction of the m6A-related clusters. **(A)** Patients were classified into m6A-related cluster A and B groups by unsupervised clustering based on the differentially expressed genes between m6A clusters. **(B)** Survival differences according to m6A-related clusters by the log-rank test. **(C)** Differential expression of 16 m6A-regulators between the m6A-related cluster A and B groups. NS, not significant; *p < 0.05; **p <0.01; ***p < 0.001; ****p < 0.0001.

To comprehensively characterize the underlying biological differences between m6A clusters, we identified DEGs between the two groups. Total 2429 DEGs were identified (adjusted p<0.01, **Table S4**). Unsupervised analysis was performed again to divide patients into distinct subgroups based on the 2429 DEGs, named m6A-related clusters A and B (**Figure 2A**). Patients with m6A-related cluster A experienced worse outcomes as compared to the ones with m6A-related cluster B (p=0.0096; **Figure 2B**). The expression level of 16 m6A-regulators was clearly different between the two m6A-related clusters (**Figure 2C**). These findings suggest the existence of m6A-regulator expression signature that plays an important role in FL.

### Generation of the m6A Score

**Figure 3:**
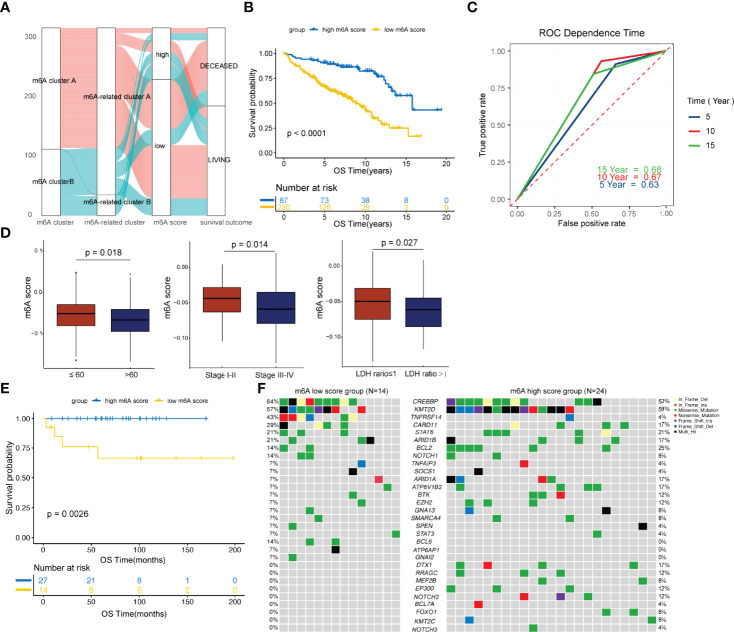
Development and validation of the m6A score. **(A)** Alluvial diagram showing the changes in m6A clusters, m6A-related clusters and m6A scores. **(B)** Comparison of survival curves between the high and low m6A score groups by the log-rank test. **(C)** The predictive value of the m6A score for survival evaluated by time-dependent ROC curves. **(D)** Differences in the m6A score among distinct clinical subgroups. **(E)** Validation of the prognostic value of the m6A score in an external cohort. **(F)** The waterfall plot depicted tumor somatic mutations of patients with low and high m6A scores in the external cohort.

To facilitate practical application, a scoring model was developed to quantify the m6A-regulator expression signature in FL patients, termed the m6A score. By using a random forest approach and univariate Cox regression analysis, twenty genes that were most relevant to m6A clusters and with significant prognostic value were isolated to construct the m6A scoring model (**Table S5** and **Figure S4**). Patients were classified into high and low m6A score groups according to the optimal threshold value calculated by the survminer package (cutoff= -0.03). **Figure 3A** shows the attribute changes in m6A-regulator expression signatures in individual patients. m6A cluster A and m6A-related cluster A had significantly low m6A scores (**Figure S5**). Moreover, patients with low m6A scores showed markedly shorter survival as compared to the ones with high m6A scores (p<0.0001; **Figures 3B** and **S6**). Patients with low and high m6A scores had median survival durations of 8.84 (95% confidence interval [CI]: 7.251-10.429) and 15.73 (95% CI: 11.729-19.731) years, respectively. Among the 20 genes used to construct the m6A scoring model, genes such as ARAP1, RNF219 and SCFD1 were inclined to be expressed in patients with low m6A scores, while genes such as MCM6, PCNT and SNX2 were enriched in patients with high m6A scores (**Figure S6**). The survival prediction according to the m6A score was evaluated by time-dependent ROC curve analysis. The area under the ROC curve for 5, 10 and 15-year survival was 0.63, 0.67 and 0.68, respectively (**Figure 3C**). In addition, it was found that age older than 60 years, advanced disease and a high LDH level were greatly linked to a low m6A score (**Figure 3D**). Multivariate Cox regression analysis demonstrated that the m6A score is an independent element of risk for FL (hazard ratio=2.757, 95% CI: 1.338-5.682, p=0.006; **Table S6**). When the cohort GSE16131 and GSE119214 were analyzed separately, we found that the m6A score still had strong potency for predicting prognosis regardless of the application of rituximab in FL (**Figure S7**).

### Validation of the m6A Score in an External Cohort

The prognostic value of m6A score was further validated in an external cohort from the Tianjin Medical University Cancer Institute and Hospital. As shown in **Figure 3E**, patients with low m6A scores showed significantly poorer survival compared to the ones with high m6A scores (p=0.0026). We then compared the genetic mutations of patients with low and high m6A scores in our own cohort. Genes such as CREBBP, TNFRSF14 and CARD11 were more frequently altered in the low m6A score group, while genes such as BCL2, ARID1A and ATP6V1B2 were greatly and often times mutated in the high m6A score group (**Figure 3F**). In addition, BCL6, ATP6AP1 and GNAI2 carried much frequent mutations in patients with low m6A scores, but in none of the patients with high m6A scores. Genes such as DTX1, RRAGC, MEF2B, EP300, NOTCH2, BCL7A, FOXO1, KMT2C and NOTCH3 were exclusively mutated in the high m6A score group (**Figure 3F**). Of note, research studies have shown that CREBBP is a key transcriptional regulator of Treg differentiation (37) and HVEM delivers a co-inhibitory signal to T cells through binding to BTLA (38), both these suggesting frequently mutated genes in the low m6A score group may mediate negatively regulate immune response.

### Differences in Infiltrating Immune Cells Between the m6A Score Groups

**Figure 4:**
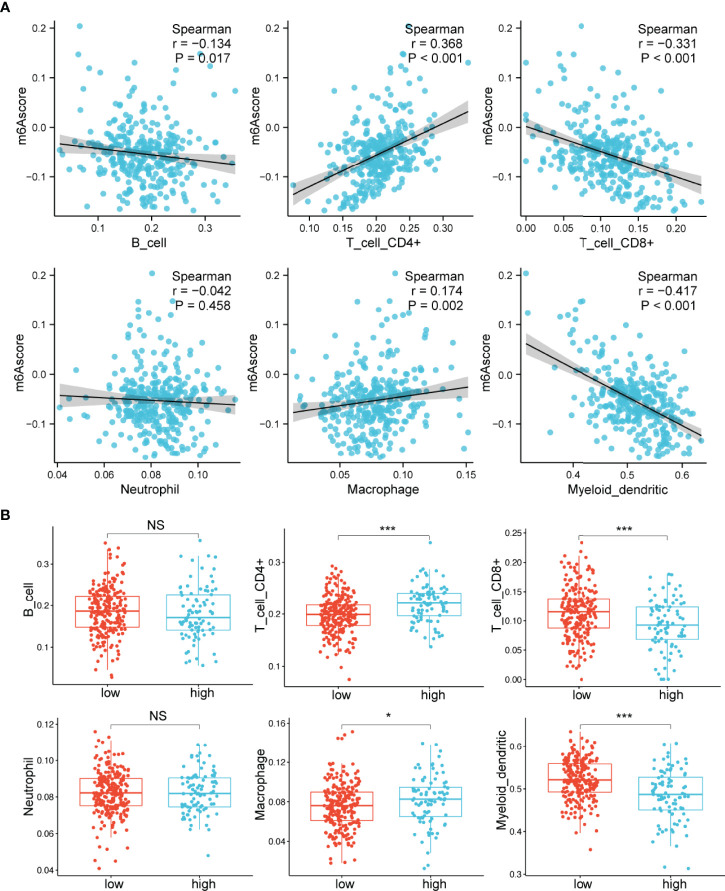
Relationships between the m6A score and infiltrating immune cells. **(A)** Correlations between the m6A score and the degree of immune cell infiltration by Spearman analysis. **(B)** Comparison of the relative abundance of infiltrating immune cells between the high and low m6A score groups. NS, not significant; *p < 0.05; ***p < 0.001.

Previous studies have demonstrated that m6A modification plays important roles in the TME (18–20). We then specifically examined the connection between the m6A score and the immune cells’ infiltrating levels. The relative abundance of six main immune cell subtypes was evaluated by TIMER (32). We found that the m6A scores were proportional to the extent of infiltration of macrophages (p=0.002) and CD4^+^ T cells (p<0.001) (**Figure 4A**). However, the m6A scores displayed a markedly inverse correlation with the infiltrationin levels of myeloid dendritic cells (p<0.001) and CD8^+^ T cells (p<0.001) (**Figure 4A**). Furthermore, the high m6A score group was observably enriched in infiltrating macrophages and CD4^+^ T cells, while the low m6A score group was prominently enriched in infiltrating myeloid dendritic cells and CD8^+^ T cells (**Figure 4B**). These findings suggested that the m6A score could be used to differentiate between groups with different immune cell infiltration patterns.

### The m6A Score Identified a Subset of Patients Harboring an Exhausted Immune Microenvironment

**Figure 5:**
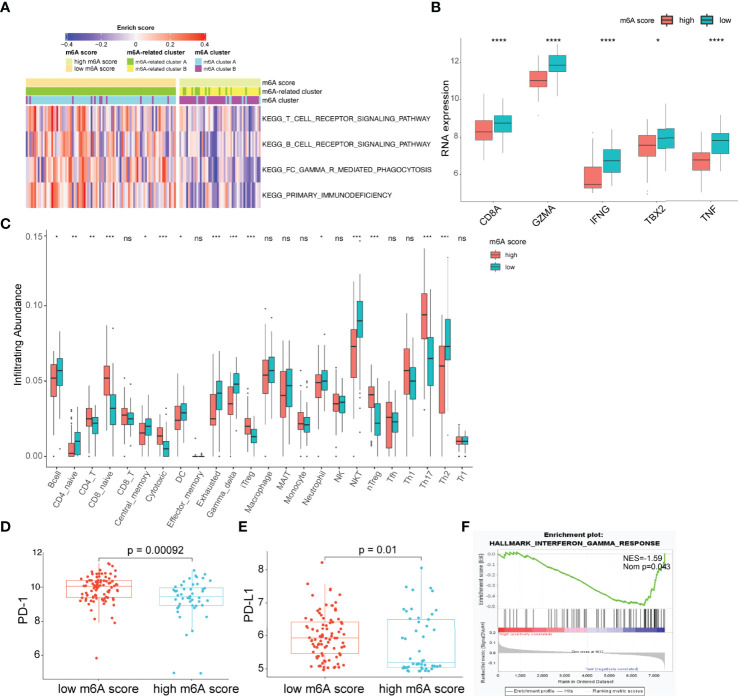
Characteristics of the TME between the high and low m6A score groups. **(A)** Heatmap showing differences in the immune-associated biological pathways between m6A score groups (adjusted p-value<0.05). **(B)** Differences in inflammatory/immune response-associated genes between m6A score groups. **(C)** Differences in the relative abundance of infiltrating immune cells between m6A score groups. **(D, E)** Differences in the expression of PD-1 **(D)** and PD-L1 **(E)** between m6A score groups. **(F)** The interferon-gamma response is enriched in the low m6A score group by GSEA. TME, tumor microenvironment; NES, normalized enrichment score; NOM p, nominal p-value; NS, not significant; *p < 0.05; **p < 0.01; ***p < 0.001; ****p < 0.0001.

The observation that patients with low m6A scores presented high infiltration of CD8^+^ T cells but had inferior OS was particularly intriguing, as increased infiltration levels of CD8^+^ T cells are usually linked to good survival. We hypothesized that these CD8^+^ T cells were exhausted T cells. The expression of PD-1 is the hallmark of exhausted T cells (39). Considering the lack of PD-1 expression data in the GSE16131 cohort, we chose the GSE119214 cohort to further analyze the immune characteristics. First, we evaluated the differences in immune responses between the high and low m6A score groups. GSVA showed that immune-associated biological processes, including T-cell receptor signaling, B-cell receptor signaling and FC gamma R-mediated phagocytosis, were significantly enhanced in patients with low m6A scores (adjusted p<0.05; **Figure 5A**). CD8A, GZMB, IFNG, TBX2 and TNF were considered to be involved in the immune/inflammatory responses according to published literature (19). A significantly upregulated expression of these genes was observed in the low m6A score group (**Figure 5B**). T cells constitute an important component of the antitumor immune cell brigade. We then used ImmuCellAI (33) to evaluate the infiltration degree of distinct T cell subtypes. As expected, we found that exhausted T cells were markedly enriched in the low m6A score group (**Figure 5C**). Moreover, the expression of PD-1 and PD-L1 in the low m6A score group was significantly increased than that in the high m6A score group (**Figures 5D, E**). In addition, GSEA demonstrates that the low m6A score group showed an enhanced IFN-γ response (**Figure 5F**). Persistent activation of IFN-γ signaling can directly upregulate the expression of PD-L1 and activate the PD-1/PD-L1 signaling axis (40). Considered as a whole, the above results indicate that the immune and inflammatory responses were enhanced in the low m6A score group. However, the expression of PD-1 and PD-L1 was also upregulated in this group and induced an exhausted immune microenvironment, ultimately leading to a poor prognosis in FL patients.

### Predicting the Response to Anti-PD-L1 Therapy Using the m6A Score

**Figure 6:**
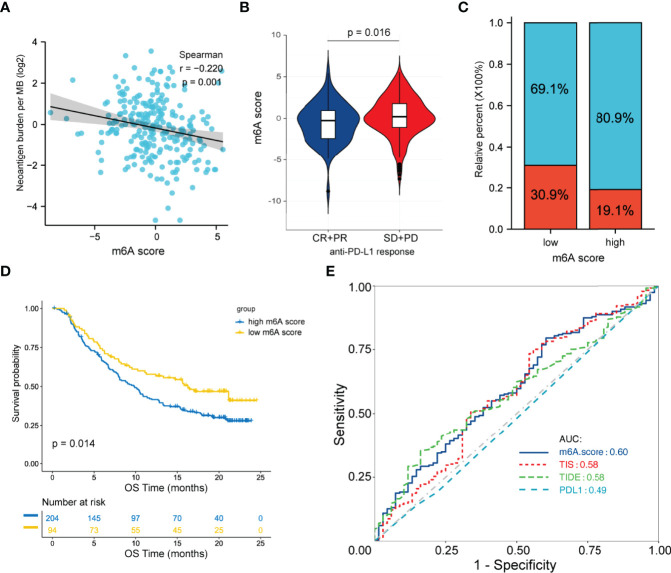
Predictive value of the m6A score for anti-PD-L1 therapy in the IMvigor210CoreBiologies cohort. **(A)** Correlation between the m6A score and tumor neoantigen burden by Spearman analysis. **(B)** Differences in the m6A score between patients with different clinical responses. **(C)** Proportion of patients with clinical response in the low and high m6A score groups. **(D)** Survival analysis between m6A score groups by the log-rank test. **(E)** ROC curve analysis for m6A score, TIDE, TIS and PD-L1 expression on tumor cells in predicting the response to anti-PD-L1 therapy. ROC, receiver operating characteristic; TIDE, tumor immune dysfunction and exclusion; TIS, tumor inflammation signature.

Immunotherapies with anti-PD-L1 antibodies have shown great success in many types of cancer. Patients with abundant CD8^+^ T cell infiltration as well as high PD-1/PD-L1 expression are more likely to benefit from anti-PD-L1 therapy. Herein, we hypothesized that the m6A score may predict a patient’s response to anti-PD-L1 therapy. Given the lack of available data on FL patients receiving anti-PD-L1 therapy, we chose an external anti-PD-L1 cohort (IMvigor210) to explore the potential predictive value of the m6A score. Of the 354 patients, 298 were evaluated for an objective response. The detailed clinical information of patients and m6A scores calculated were showed in **Table S7**. We found that the m6A score presented a significant negative correlation with tumor neoantigen burden (p=0.001, **Figure 6A**). Moreover, low m6A score group displayed significantly clinical and survival benefits than high m6A score group (**Figures 6B–D**). Finally, we compared the performance of m6A score with other predictive biomarkers. We found that the m6A score has a slight advantage over TIS and TIDE. The area under the ROC curve for m6A score, TIDE, TIS and PD-L1 expression on tumor cells was 0.60, 0.58, 0.58 and 0.49 respectively (**Figure 6E**).

## Discussion

Epigenetic deregulation is critical to the tumorigenesis of FL (4, 5). Genetic alterations involved in the posttranslational modification of histones, such as the histone methyltransferases KMT2D and KMT2C and the histone acetyltransferases CREBBP and EP300, frequently occur in FL (4). However, the role of m6A methylation, as the most frequent posttranscriptional mRNA modification, is less known in FL.

Specific m6A-regulators have specific carcinogenic properties that vary in various tumors. Some serve as tumor inhibitors, but others serve as tumor promoters. Hence, an integrated and comprehensive analysis of m6A-regulators is needed. In this study, we identified two m6A clusters, which was significantly associated with distinct carcinogenic processes and could be used to predict the prognosis of FL patients. Moreover, m6A-related clusters were constructed to comprehensively characterize the underlying biological differences between m6A clusters. The m6A score, a quantification model, was further developed to reflect the synthetic cross-talk of m6A modification. Low m6A scores were significantly associated with poorer patient survival than high m6A scores.

The role of m6A modification in the TME has been largely investigated (18–20). m6A-regulators showed either a positive or negative correlation with the level of immune cell infiltration regardless of their categorization as a “writer”, “reader”, or “eraser” (19). The TME usually represents a major obstacle that restricts the anti-tumor immune responses and limits the efficacy of immunotherapeutic agents. FL is characterized by high infiltration of T-cell subpopulations (5). Here, we observed that patients with high m6A scores had enhanced CD4^+^ T cell infiltration, and those with low m6A scores had enhanced CD8^+^ T cell infiltration. Moreover, the low m6A score group harbored enriched immune and inflammatory responses. Nevertheless, PD-1/PD-L1 signaling was also activated and subverted the antitumor response of cytotoxic CD8^+^ T cells, which promoted immune escape. Ultimately, this exhausted TME led to an inferior prognosis in patients with low m6A scores.

PD-1/PD-L1 signaling blockade reverses local immunosuppression. Clinical responses to PD-1/PD-L1 inhibitors occur most often in patients with rich but exhausted immune cell infiltration (41–43). Patients with low m6A scores represent a subset of candidates who might benefit from anti-PD-1/PD-L1 immunotherapy. Anti-PD-1 antibody response rates in unselected FL patients have been lower than expected (44). Our study first uncovered the prediction of immunotherapy response that could be attributed to m6A modification in FL. What is worth mentioning is that the m6A score shows effective predictive ability in immune response in a solid tumor cohort, although it was developed from FL patients. Hence, the application of m6A score in predicting immune efficacy may be extended to pan-cancers. More studies are urgently needed to validate this finding.

m6A modification is a reversible and dynamic process; targeting m6A-regulators to change m6A modification patterns and further reshaping the adverse cell infiltration characteristics in TME may enhance the efficacy of immunotherapy. A preclinical study by Han D and colleagues showed that Ythdf1-deficient mice presented an elevated CD8^+^ T cell antitumor immune response, and the effectiveness of PD-L1 inhibition therapy was improved in these mice. This result indicates that YTHDF1 is a potential treatment target and that combination therapies can increase immunotherapy efficacy (45). A variety of small-molecule drugs targeting m6A-regulators have been studied, although the clinical applications of these drugs still need further development (46–48). Our study provides a new insight into the combination therapy strategies targeting m6A modification and immunotherapy to improve FL patients’ clinical responses.

In conclusion, our study demonstrated that m6A-regulator expression signatures were characterized by significant differences in the immune landscape of FL. The m6A score identified a subset of FL harboring an exhausted tumor microenvironment and may contribute to personalized immunotherapy strategies making in patients with FL.

## Data Availability Statement

The original contributions presented in the study are publicly available. This data can be found here: https://db.cngb.org/search/project/CNP0002472/ and https://db.cngb.org/search/project/CNP0002473/.

## Ethics Statement

The studies involving human participants were reviewed and approved by the Clinical Research Ethics Board of the Tianjin Medical University Cancer Institute and Hospital. The patients/participants provided their written informed consent to participate in this study.

## Author Contributions

XW conceived and designed the study. XW and HZ supervised all aspects of the research project and interpreted the data. TZ and HL performed the research and statistical and bioinformatics analyses. TZ, FG, and YC collected data and prepared the images. JH, LL, LQ, ZQ, and SZ analyzed clinical information. WG and BM provided some suggestions about FL classifications. XR provided technical support. TZ wrote the manuscript and finalized the figures. XW and HZ reviewed the manuscript. All authors contributed to the article and approved the submitted version.

## Funding

This study was supported by grants from the Natural Science Foundation of Tianjin (19JCYBJC26500), the National Natural Science Foundation of China (81770213), the Clinical Oncology Research Fund of CSCO (Y-XD2019-162, Y-Roche20192-0097), and the National Human Genetic Resources Sharing Service Platform/Cancer Biobank of Tianjin Medical University Cancer Institute and Hospital grant (2005DKA21300).

## Conflict of Interest

The authors declare that the research was conducted in the absence of any commercial or financial relationships that could be construed as a potential conflict of interest.

## Publisher’s Note

All claims expressed in this article are solely those of the authors and do not necessarily represent those of their affiliated organizations, or those of the publisher, the editors and the reviewers. Any product that may be evaluated in this article, or claim that may be made by its manufacturer, is not guaranteed or endorsed by the publisher.

## Supplementary Material

Supplementary Figure 1 Flowchart of the study design. FL, follicular lymphoma; DLBCL, diffuse large B cell lymphoma; OS, overall survival; TMUCH, Tianjin Medical University Cancer Institute and Hospital.

Supplementary Figure 2 The interactions among 16 m6A-regulators in follicular lymphoma. **(A)** Chromosomal distribution of 16 m6A-regulator genes. **(B)** Pairwise correlation of 16 m6A-regulators by Spearman analysis. Red (blue) indicates positive (negative) correlations. *p < 0.05; **p < 0.01; ***p < 0.001. **(C)** Protein-protein interaction among the 16 m6A-regulators.

Supplementary Figure 3 Identification of consensus clusters by 16 m6A-regulators. **(A)** Consensus clustering matrices for k = 3 to 10. **(B)** Relative change in the area under the cumulative distribution function curve.

Supplementary Figure 4 Forest plot shows the 20 prognostic genes used to construct the m6A scoring model.

Supplementary Figure 5 Distribution of the m6A score between the m6A clusters and m6A-related clusters. ****p < 0.0001.

Supplementary Figure 6 Distribution of the m6A score, overall survival, overall survival status and heatmap of the 20 prognostic genes used to construct the m6A scoring model.

Supplementary Figure 7 The prognostic value of the m6A score in the cohort GES16131 **(A)** and GSE119214 **(B)**.
