# Supplementary material for: m6A-Regulator Expression Signatures Identify a Subset of Follicular Lymphoma Harboring an Exhausted Tumor Microenvironment
Source: Front Immunol. 2022 Jun 6;13:922471. doi: 10.3389/fimmu.2022.922471 (PMC9207509; doi:10.3389/fimmu.2022.922471)
Supplement: Supplementary file 1 [file DataSheet_1.docx]

| **Dataset** | **GSE16131** | **GSE119214** | **GSE66166** |
| --- | --- | --- | --- |
| **Number of samples** | 184 | 137 | 30* |
| **Number of samples with survival data** | 180 | 137 | 0 |
| **Time of biopsy** | at diagnosis | at diagnosis | at diagnosis |
| **Treatment** | standard treatments  (observation, chemotherapy, et al. ) | Immunochemotherapy | Immunochemotherapy |
| **Rituximab** | No | Yes | Yes |
| **Grade** | 1-3a | 1-3a | 1-3a |

**Table S1. Summary of the clinical information of the datasets used in this study**

* GSE66166 included 138 samples, 108 of which have already been included in GSE119214. Hence, only remaining 30 samples were enrolled for further analysis.

**Table S2. Correlation of the 16 m6A-regulators by Spearman analysis**

| **From** | **To** | **Correlation** | **p Value** |
| --- | --- | --- | --- |
| HNRNPC | HNRNPA2B1 | 0.149018616 | 0.007870522 |
| HNRNPC | METTL14 | -0.177654309 | 0.001493985 |
| HNRNPC | WTAP | -0.138037826 | 0.013902567 |
| HNRNPC | ELAVL1 | 0.265084887 | 1.69E-06 |
| HNRNPC | ALKBH5 | 0.285849256 | 2.25E-07 |
| HNRNPC | YTHDC1 | 0.121166742 | 0.031025731 |
| HNRNPC | LRPPRC | 0.269346967 | 1.13E-06 |
| HNRNPC | RBM15 | 0.232005911 | 3.03E-05 |
| HNRNPC | METTL3 | 0.119459679 | 0.033491929 |
| YTHDF2 | KIAA1429 | 0.135071611 | 0.016109766 |
| YTHDF2 | METTL14 | 0.124174049 | 0.027057659 |
| KIAA1429 | IGF2BP1 | -0.123812407 | 0.027510523 |
| KIAA1429 | HNRNPA2B1 | 0.111741463 | 0.046826014 |
| KIAA1429 | CBLL1 | 0.16382832 | 0.003442945 |
| KIAA1429 | ELAVL1 | -0.110263135 | 0.049831388 |
| KIAA1429 | LRPPRC | 0.123074077 | 0.028455329 |
| IGF2BP1 | HNRNPA2B1 | -0.157099183 | 0.005055906 |
| IGF2BP1 | ZC3H13 | 0.273145405 | 7.88E-07 |
| IGF2BP1 | METTL14 | 0.11888431 | 0.034359924 |
| IGF2BP1 | WTAP | 0.126676351 | 0.024096163 |
| IGF2BP1 | ALKBH5 | -0.152464392 | 0.00653321 |
| IGF2BP1 | YTHDC1 | -0.138643901 | 0.013485781 |
| IGF2BP1 | LRPPRC | -0.213806457 | 0.000124989 |
| IGF2BP1 | METTL3 | -0.136045285 | 0.015353625 |
| HNRNPA2B1 | CBLL1 | 0.175201847 | 0.001740272 |
| HNRNPA2B1 | ZC3H13 | -0.124124888 | 0.027118842 |
| HNRNPA2B1 | ELAVL1 | 0.203002294 | 0.000274735 |
| HNRNPA2B1 | LRPPRC | 0.234181059 | 2.53E-05 |
| HNRNPA2B1 | METTL3 | 0.21601632 | 0.000105867 |
| RBM15B | ALKBH5 | 0.134192406 | 0.016820331 |
| RBM15B | YTHDC1 | 0.11043415 | 0.049475689 |
| CBLL1 | ZC3H13 | 0.187520819 | 0.000792683 |
| CBLL1 | METTL14 | 0.114184971 | 0.042191761 |
| CBLL1 | WTAP | 0.15820195 | 0.004752025 |
| CBLL1 | ELAVL1 | 0.137044281 | 0.014610315 |
| CBLL1 | ALKBH5 | -0.148415669 | 0.008128129 |
| ZC3H13 | METTL14 | 0.26454791 | 1.78E-06 |
| ZC3H13 | WTAP | 0.203165655 | 0.000271564 |
| ZC3H13 | ALKBH5 | -0.289401202 | 1.56E-07 |
| ZC3H13 | YTHDC1 | -0.149765719 | 0.007561438 |
| ZC3H13 | LRPPRC | -0.118376954 | 0.035141117 |
| ZC3H13 | METTL3 | -0.177855387 | 0.001475282 |
| METTL14 | WTAP | 0.137975603 | 0.01394599 |
| METTL14 | ELAVL1 | -0.159167431 | 0.004499591 |
| METTL14 | ALKBH5 | -0.273380738 | 7.71E-07 |
| WTAP | ALKBH5 | -0.250978978 | 6.08E-06 |
| WTAP | RBM15 | -0.16187138 | 0.003855679 |
| ELAVL1 | ALKBH5 | 0.336307344 | 8.05E-10 |
| ELAVL1 | LRPPRC | 0.214962537 | 0.000114614 |
| ELAVL1 | METTL3 | 0.217069371 | 9.78E-05 |
| ALKBH5 | YTHDC1 | 0.238151238 | 1.83E-05 |
| ALKBH5 | LRPPRC | 0.304473963 | 3.18E-08 |
| ALKBH5 | RBM15 | 0.210829011 | 0.000155908 |
| ALKBH5 | METTL3 | 0.148530326 | 0.008078571 |
| YTHDC1 | METTL3 | 0.142566133 | 0.011044608 |
| LRPPRC | RBM15 | 0.169172554 | 0.002511574 |
| LRPPRC | METTL3 | 0.122978978 | 0.028579022 |

**Table S3. Comparison of activation states of biological pathways between m6A clusters A and B by GSVA**

| **pathway** | **p** | **p.adj** | **method** |
| --- | --- | --- | --- |
| KEGG_GLYCOSPHINGOLIPID_BIOSYNTHESIS_GLOBO_SERIES | 9.77E-10 | 5.00E-08 | Wilcoxon |
| KEGG_NEUROACTIVE_LIGAND_RECEPTOR_INTERACTION | 1.07E-09 | 5.00E-08 | Wilcoxon |
| KEGG_CELL_CYCLE | 6.13E-10 | 5.00E-08 | Wilcoxon |
| KEGG_HEDGEHOG_SIGNALING_PATHWAY | 3.62E-10 | 5.00E-08 | Wilcoxon |
| KEGG_ADIPOCYTOKINE_SIGNALING_PATHWAY | 1.04E-08 | 3.90E-07 | Wilcoxon |
| KEGG_PEROXISOME | 2.60E-08 | 6.90E-07 | Wilcoxon |
| KEGG_HYPERTROPHIC_CARDIOMYOPATHY_HCM | 2.35E-08 | 6.90E-07 | Wilcoxon |
| KEGG_CALCIUM_SIGNALING_PATHWAY | 4.90E-08 | 1.10E-06 | Wilcoxon |
| KEGG_ARRHYTHMOGENIC_RIGHT_VENTRICULAR_CARDIOMYOPATHY_ARVC | 2.39E-07 | 4.40E-06 | Wilcoxon |
| KEGG_DILATED_CARDIOMYOPATHY | 2.24E-07 | 4.40E-06 | Wilcoxon |
| KEGG_T_CELL_RECEPTOR_SIGNALING_PATHWAY | 3.86E-07 | 6.00E-06 | Wilcoxon |
| KEGG_PATHWAYS_IN_CANCER | 3.84E-07 | 6.00E-06 | Wilcoxon |
| KEGG_DNA_REPLICATION | 4.73E-07 | 6.80E-06 | Wilcoxon |
| KEGG_PPAR_SIGNALING_PATHWAY | 5.18E-07 | 6.90E-06 | Wilcoxon |
| KEGG_UBIQUITIN_MEDIATED_PROTEOLYSIS | 3.00E-06 | 3.70E-05 | Wilcoxon |
| KEGG_FRUCTOSE_AND_MANNOSE_METABOLISM | 3.35E-06 | 3.90E-05 | Wilcoxon |
| KEGG_GLYCOSPHINGOLIPID_BIOSYNTHESIS_GANGLIO_SERIES | 3.89E-06 | 4.30E-05 | Wilcoxon |
| KEGG_ONE_CARBON_POOL_BY_FOLATE | 4.26E-06 | 4.40E-05 | Wilcoxon |
| KEGG_GNRH_SIGNALING_PATHWAY | 5.26E-06 | 5.10E-05 | Wilcoxon |
| KEGG_BASAL_CELL_CARCINOMA | 5.49E-06 | 5.10E-05 | Wilcoxon |
| KEGG_FOCAL_ADHESION | 5.87E-06 | 5.20E-05 | Wilcoxon |
| KEGG_N_GLYCAN_BIOSYNTHESIS | 9.59E-06 | 8.10E-05 | Wilcoxon |
| KEGG_OOCYTE_MEIOSIS | 1.27E-05 | 1.00E-04 | Wilcoxon |
| KEGG_RENAL_CELL_CARCINOMA | 1.94E-05 | 0.00015 | Wilcoxon |
| KEGG_RNA_DEGRADATION | 2.83E-05 | 0.00021 | Wilcoxon |
| KEGG_MAPK_SIGNALING_PATHWAY | 4.21E-05 | 3.00E-04 | Wilcoxon |
| KEGG_CYTOKINE_CYTOKINE_RECEPTOR_INTERACTION | 5.02E-05 | 0.00035 | Wilcoxon |
| KEGG_VIBRIO_CHOLERAE_INFECTION | 5.52E-05 | 0.00037 | Wilcoxon |
| KEGG_RIBOFLAVIN_METABOLISM | 6.65E-05 | 0.00043 | Wilcoxon |
| KEGG_NITROGEN_METABOLISM | 0.000126294 | 0.00078 | Wilcoxon |
| KEGG_NOD_LIKE_RECEPTOR_SIGNALING_PATHWAY | 0.000130026 | 0.00078 | Wilcoxon |
| KEGG_PRIMARY_IMMUNODEFICIENCY | 0.000137191 | 8.00E-04 | Wilcoxon |
| KEGG_PROSTATE_CANCER | 0.000158159 | 0.00089 | Wilcoxon |
| KEGG_APOPTOSIS | 0.000171998 | 0.00092 | Wilcoxon |
| KEGG_VEGF_SIGNALING_PATHWAY | 0.00017352 | 0.00092 | Wilcoxon |
| KEGG_MTOR_SIGNALING_PATHWAY | 0.000250128 | 0.0012 | Wilcoxon |
| KEGG_FC_EPSILON_RI_SIGNALING_PATHWAY | 0.000244798 | 0.0012 | Wilcoxon |
| KEGG_MELANOGENESIS | 0.000246917 | 0.0012 | Wilcoxon |
| KEGG_AXON_GUIDANCE | 0.000265637 | 0.0013 | Wilcoxon |
| KEGG_ACUTE_MYELOID_LEUKEMIA | 0.000289362 | 0.0013 | Wilcoxon |
| KEGG_PORPHYRIN_AND_CHLOROPHYLL_METABOLISM | 0.000369877 | 0.0017 | Wilcoxon |
| KEGG_SELENOAMINO_ACID_METABOLISM | 0.000421068 | 0.0019 | Wilcoxon |
| KEGG_PROTEASOME | 0.000455638 | 0.002 | Wilcoxon |
| KEGG_MELANOMA | 0.000575894 | 0.0024 | Wilcoxon |
| KEGG_P53_SIGNALING_PATHWAY | 0.000707928 | 0.0029 | Wilcoxon |
| KEGG_WNT_SIGNALING_PATHWAY | 0.000713638 | 0.0029 | Wilcoxon |
| KEGG_BASE_EXCISION_REPAIR | 0.000785586 | 0.0031 | Wilcoxon |
| KEGG_GLYCOSAMINOGLYCAN_BIOSYNTHESIS_KERATAN_SULFATE | 0.000881494 | 0.0034 | Wilcoxon |
| KEGG_MISMATCH_REPAIR | 0.000935257 | 0.0036 | Wilcoxon |
| KEGG_LONG_TERM_POTENTIATION | 0.001115314 | 0.0041 | Wilcoxon |
| KEGG_VIRAL_MYOCARDITIS | 0.00113278 | 0.0041 | Wilcoxon |
| KEGG_SPLICEOSOME | 0.001332071 | 0.0047 | Wilcoxon |
| KEGG_GAP_JUNCTION | 0.001306755 | 0.0047 | Wilcoxon |
| KEGG_OLFACTORY_TRANSDUCTION | 0.001400024 | 0.0048 | Wilcoxon |
| KEGG_SULFUR_METABOLISM | 0.001557373 | 0.0052 | Wilcoxon |
| KEGG_VASCULAR_SMOOTH_MUSCLE_CONTRACTION | 0.001563283 | 0.0052 | Wilcoxon |
| KEGG_OXIDATIVE_PHOSPHORYLATION | 0.001692257 | 0.0055 | Wilcoxon |
| KEGG_TYPE_II_DIABETES_MELLITUS | 0.001737378 | 0.0055 | Wilcoxon |
| KEGG_ENDOMETRIAL_CANCER | 0.001724375 | 0.0055 | Wilcoxon |
| KEGG_HUNTINGTONS_DISEASE | 0.001844696 | 0.0057 | Wilcoxon |
| KEGG_CHRONIC_MYELOID_LEUKEMIA | 0.001900608 | 0.0058 | Wilcoxon |
| KEGG_OTHER_GLYCAN_DEGRADATION | 0.002140174 | 0.0063 | Wilcoxon |
| KEGG_REGULATION_OF_ACTIN_CYTOSKELETON | 0.002140174 | 0.0063 | Wilcoxon |
| KEGG_TAURINE_AND_HYPOTAURINE_METABOLISM | 0.002478487 | 0.0071 | Wilcoxon |
| KEGG_NATURAL_KILLER_CELL_MEDIATED_CYTOTOXICITY | 0.002496623 | 0.0071 | Wilcoxon |
| KEGG_STEROID_HORMONE_BIOSYNTHESIS | 0.00266539 | 0.0075 | Wilcoxon |
| KEGG_JAK_STAT_SIGNALING_PATHWAY | 0.002704282 | 0.0075 | Wilcoxon |
| KEGG_ENDOCYTOSIS | 0.003224652 | 0.0088 | Wilcoxon |
| KEGG_GLYCEROLIPID_METABOLISM | 0.003377248 | 0.0091 | Wilcoxon |
| KEGG_RIBOSOME | 0.004098288 | 0.011 | Wilcoxon |
| KEGG_PROTEIN_EXPORT | 0.004675229 | 0.012 | Wilcoxon |
| KEGG_ALANINE_ASPARTATE_AND_GLUTAMATE_METABOLISM | 0.005416021 | 0.014 | Wilcoxon |
| KEGG_BASAL_TRANSCRIPTION_FACTORS | 0.005993947 | 0.015 | Wilcoxon |
| KEGG_SMALL_CELL_LUNG_CANCER | 0.006136549 | 0.015 | Wilcoxon |
| KEGG_ARACHIDONIC_ACID_METABOLISM | 0.006715642 | 0.017 | Wilcoxon |
| KEGG_SPHINGOLIPID_METABOLISM | 0.006919319 | 0.017 | Wilcoxon |
| KEGG_RIG_I_LIKE_RECEPTOR_SIGNALING_PATHWAY | 0.007689202 | 0.019 | Wilcoxon |
| KEGG_O_GLYCAN_BIOSYNTHESIS | 0.008702534 | 0.02 | Wilcoxon |
| KEGG_LEISHMANIA_INFECTION | 0.008370338 | 0.02 | Wilcoxon |
| KEGG_CARDIAC_MUSCLE_CONTRACTION | 0.00937234 | 0.022 | Wilcoxon |
| KEGG_GLYCOSYLPHOSPHATIDYLINOSITOL_GPI_ANCHOR_BIOSYNTHESIS | 0.011269888 | 0.025 | Wilcoxon |
| KEGG_PARKINSONS_DISEASE | 0.010989342 | 0.025 | Wilcoxon |
| KEGG_AMYOTROPHIC_LATERAL_SCLEROSIS_ALS | 0.010681161 | 0.025 | Wilcoxon |
| KEGG_GLYCOLYSIS_GLUCONEOGENESIS | 0.014356476 | 0.031 | Wilcoxon |
| KEGG_AMINO_SUGAR_AND_NUCLEOTIDE_SUGAR_METABOLISM | 0.01400877 | 0.031 | Wilcoxon |
| KEGG_PHOSPHATIDYLINOSITOL_SIGNALING_SYSTEM | 0.015872516 | 0.034 | Wilcoxon |
| KEGG_HEMATOPOIETIC_CELL_LINEAGE | 0.015872516 | 0.034 | Wilcoxon |
| KEGG_INOSITOL_PHOSPHATE_METABOLISM | 0.016606976 | 0.035 | Wilcoxon |
| KEGG_CIRCADIAN_RHYTHM_MAMMAL | 0.016959821 | 0.035 | Wilcoxon |
| KEGG_GLYCOSAMINOGLYCAN_DEGRADATION | 0.017267479 | 0.036 | Wilcoxon |
| KEGG_ALDOSTERONE_REGULATED_SODIUM_REABSORPTION | 0.018824499 | 0.038 | Wilcoxon |
| KEGG_LEUKOCYTE_TRANSENDOTHELIAL_MIGRATION | 0.023365839 | 0.047 | Wilcoxon |

**Table S4. Significantly differentially expressed genes between m6A cluster A and m6A cluster B groups**

| A1BG | ASXL2 | CAMSAP1 | CPNE2 | EEF2 | FOXK1 | HSPA1L | LDLRAD3 | MPZL2 | OAS3 | PPIL3 | RILP | SIX4 | SUMF2 | TOR1AIP1 | WDR60 |
| --- | --- | --- | --- | --- | --- | --- | --- | --- | --- | --- | --- | --- | --- | --- | --- |
| AAGAB | ATAD1 | CAPS | CPPED1 | EEFSEC | FOXN2 | HTR7 | LDOC1L | MRAS | OAZ1 | PPM1D | RIMS2 | SKAP2 | SUPT16H | TOR1AIP2 | WDR78 |
| AASDH | ATAD2B | CAPS2 | CPSF2 | EFNA5 | FOXO1 | HTRA3 | LEMD2 | MREG | OBP2A | PPM1M | RIMS4 | SKIV2L2 | SURF6 | TOR3A | WDR81 |
| ABCB1 | ATAD3C | CARD11 | CPSF3 | EFR3B | FOXP3 | IDH3A | LENG1 | MRFAP1 | OCIAD2 | PPME1 | RIN2 | SLAIN1 | SUSD3 | TP53AIP1 | WDR86 |
| ABCB10 | ATF6 | CARD14 | CPT1C | EGOT | FOXRED1 | IER3IP1 | LEO1 | MRPL1 | OCLN | PPOX | RIOK1 | SLAMF7 | SUV420H2 | TPBG | WDSUB1 |
| ABCC11 | ATF7 | CARD18 | CPXM2 | EGR1 | FPR3 | IFI27L1 | LEPREL1 | MRPL10 | OFD1 | PPP1R12C | RIPK4 | SLC12A9 | SVEP1 | TPCN2 | WFDC3 |
| ABCC13 | ATF7IP | CARD8 | CRB1 | EHF | FRMD8 | IFI27L2 | LEPROT | MRPL14 | OGFRL1 | PPP1R14A | RLTPR | SLC13A2 | SVOP | TPD52L3 | WHSC1L1 |
| ABCC3 | ATF7IP2 | CARS | CRBN | EID3 | FRZB | IFIT2 | LETM1 | MRPL2 | OMA1 | PPP1R16B | RNASE11 | SLC15A4 | SYCN | TPM1 | WIPF2 |
| ABCC4 | ATG16L2 | CARS2 | CRIM1 | EIF1 | FSTL1 | IFIT3 | LHFP | MRPL20 | OPA1 | PPP1R3B | RNASEH1 | SLC19A1 | SYNE2 | TPM3 | WNK1 |
| ABCC9 | ATG4C | CASD1 | CRIPAK | EIF1AD | FUNDC1 | IFNGR1 | LHX6 | MRPL21 | OPALIN | PPP1R3C | RNASEH2C | SLC19A3 | SYNPO2 | TPMT | WNT8A |
| ABHD10 | ATL3 | CASP14 | CRIPT | EIF2AK2 | FXR1 | IFRD1 | LIMA1 | MRPL32 | OPN5 | PPP1R3F | RNF112 | SLC1A2 | SYS1 | TPR | WNT9A |
| ABHD14B | ATP13A4 | CATSPER3 | CRLS1 | EIF2S3 | FYB | IFT81 | LIMCH1 | MRPL35 | OPRK1 | PPP1R9B | RNF115 | SLC22A5 | SYT3 | TPRXL | WRNIP1 |
| ABHD15 | ATP1B3 | CBLB | CROT | EIF3C | FYTTD1 | IGF2BP1 | LIN37 | MRPL36 | OR51B5 | PPP2R3C | RNF123 | SLC23A3 | SYT7 | TRAF1 | WSCD2 |
| ABI2 | ATP2A2 | CBR4 | CRTC2 | EIF3K | GAB1 | IGHMBP2 | LIN54 | MRPL37 | OR51M1 | PPP2R5E | RNF149 | SLC25A15 | TACC1 | TRAF6 | XIAP |
| ABI3 | ATP5G3 | CBX4 | CRTC3 | EIF3M | GABPB2 | IGSF6 | LIN7B | MRPL38 | ORAOV1 | PPP6C | RNF157 | SLC25A19 | TACSTD2 | TRAFD1 | XPNPEP1 |
| ABI3BP | ATP6AP1L | CBX5 | CRX | EIF4E2 | GADD45GIP1 | IK | LIN9 | MRPL41 | OSBPL11 | PPTC7 | RNF175 | SLC25A2 | TAF1 | TRAP1 | XPO1 |
| ABTB1 | ATP6V0A2 | CC2D2A | CRYGS | EIF4EBP2 | GALK1 | IKZF2 | LINGO1 | MRPL50 | OSBPL5 | PPWD1 | RNF181 | SLC25A26 | TAF10 | TRDMT1 | XPO4 |
| ACAA2 | ATP6V1C1 | CCBE1 | CRYZL1 | EIF4G2 | GALK2 | IL17RA | LIPH | MRPL51 | OSBPL8 | PQLC1 | RNF183 | SLC25A28 | TAF13 | TRIB1 | XPO5 |
| ACAD10 | ATP6V1D | CCDC104 | CSTB | ELAVL1 | GALM | IL17RC | LIX1L | MRPL54 | OSMR | PRELID1 | RNF185 | SLC25A36 | TAF15 | TRIM13 | XPOT |
| ACAP3 | ATP6V1G1 | CCDC108 | CSTF3 | ELAVL3 | GALNT10 | IL18BP | LLGL1 | MRPS10 | OSTM1 | PREPL | RNF19A | SLC25A39 | TAF6L | TRIM2 | XRCC6BP1 |
| ACBD6 | ATPAF1 | CCDC113 | CTAGE5 | ELF2 | GAPVD1 | IL1RAPL1 | LLPH | MRPS15 | OTUD7B | PREX1 | RNF216 | SLC25A46 | TAF8 | TRIM35 | YIPF4 |
| ACOT8 | ATPIF1 | CCDC117 | CTNNB1 | ELK4 | GATA6 | IL20RA | LMBR1 | MRPS18C | OVOL1 | PRKAA1 | RNF219 | SLC26A6 | TAGLN | TRIM4 | YIPF5 |
| ACP6 | ATXN1 | CCDC132 | CTSB | ELMOD3 | GATAD1 | IL20RB | LMF1 | MRPS23 | OXNAD1 | PRKAB2 | RNF26 | SLC26A8 | TANK | TRIM41 | YPEL3 |
| ACPT | ATXN3 | CCDC134 | CTSC | ELOVL3 | GATAD2A | IL21R | LMNA | MRPS26 | P2RX7 | PRKCG | RNF31 | SLC27A4 | TAOK1 | TRIM46 | YPEL5 |
| ACRBP | ATXN7 | CCDC135 | CTSS | ELP3 | GATAD2B | IL34 | LOC100128164 | MRPS30 | P2RY8 | PRKCH | RNF40 | SLC28A1 | TAPT1 | TRIM47 | YTHDC1 |
| ACSS1 | ATXN7L1 | CCDC136 | CTU2 | EML4 | GATC | IL6R | LOC100129550 | MRPS31 | P4HA3 | PRKD2 | RNF41 | SLC29A4 | TARS | TRIM5 | YWHAH |
| ACSS2 | ATXN7L2 | CCDC14 | CUEDC1 | EMP1 | GATM | ILDR1 | LOC100190986 | MRRF | PACSIN2 | PRKD3 | RNPC3 | SLC2A9 | TAS2R45 | TRIM56 | YY1 |
| ACSS3 | AURKAIP1 | CCDC147 | CUL1 | ENO1 | GBP1 | ILF2 | LOC154761 | MRVI1 | PAFAH1B2 | PRKG1 | ROCK1 | SLC30A1 | TBC1D1 | TRIM6 | ZADH2 |
| ACTB | AVIL | CCDC151 | CUL7 | ENPP7 | GBP4 | INADL | LOC441204 | MS4A6A | PAN3 | PRM3 | RP9P | SLC30A2 | TBC1D15 | TRIM62 | ZAN |
| ACTR2 | AVL9 | CCDC43 | CUL9 | ENSA | GBP5 | ING1 | LOC728613 | MSL3 | PAOX | PRMT6 | RPA1 | SLC30A6 | TBC1D22B | TRIM67 | ZBED5 |
| ACTR3 | B2M | CCDC50 | CWC15 | ENTPD5 | GDE1 | INGX | LOC93622 | MSN | PAPD5 | PROKR2 | RPAIN | SLC30A7 | TBC1D2B | TRIM8 | ZBTB10 |
| ACTR6 | B3GALTL | CCDC59 | CWC22 | EPB41L1 | GDI2 | INHBA | LOH12CR2 | MSRA | PAPOLA | PROSC | RPAP2 | SLC30A9 | TBX1 | TRIP11 | ZBTB11 |
| ADAD2 | B3GNT8 | CCDC6 | CWF19L1 | EPB41L4A | GDNF | INO80 | LONRF1 | MTAP | PAPOLG | PRPF8 | RPF2 | SLC31A1 | TBX18 | TRIP12 | ZBTB2 |
| ADAM11 | B3GNT9 | CCDC64 | CXorf36 | EPB41L4B | GEMIN6 | INO80D | LOXHD1 | MTCH2 | PARL | PRR15 | RPH3AL | SLC35A3 | TCEA3 | TRNT1 | ZBTB33 |
| ADAM22 | B4GALNT4 | CCDC77 | CXorf38 | EPC1 | GEMIN8 | INPP5F | LPAR5 | MTDH | PARM1 | PRRC1 | RPL10A | SLC35A4 | TCEAL3 | TRPC4 | ZBTB37 |
| ADAM33 | BACE2 | CCDC84 | CXXC5 | EPHA4 | GFI1B | INSR | LPCAT2 | MTIF3 | PARP10 | PRRT2 | RPL15 | SLC35C1 | TCEB2 | TRPS1 | ZBTB38 |
| ADAM7 | BAD | CCDC88A | CYB561D1 | EPM2AIP1 | GFM1 | INTS10 | LPIN3 | MTMR12 | PARS2 | PSD2 | RPL18 | SLC35C2 | TCF19 | TRPT1 | ZBTB4 |
| ADAMTS1 | BAG5 | CCDC88B | CYB561D2 | EPS15L1 | GFRA1 | INTS2 | LPP | MTMR6 | PARVG | PSD4 | RPL19 | SLC35E1 | TCF21 | TRPV2 | ZBTB43 |
| ADAMTS10 | BAHCC1 | CCDC88C | CYB5D1 | EPS8L2 | GGT6 | INTS4 | LPXN | MTMR9 | PATE1 | PSEN1 | RPL24 | SLC35F1 | TCFL5 | TRUB2 | ZBTB47 |
| ADAMTS8 | BANF2 | CCDC97 | CYBB | ERCC2 | GGT7 | INTS9 | LRCH3 | MTR | PATL1 | PSMA5 | RPL27 | SLC36A4 | TCOF1 | TSC22D2 | ZBTB8OS |
| ADAMTSL5 | BANP | CCL26 | CYP19A1 | ERF | GHRL | IPMK | LRFN4 | MTRF1 | PAWR | PSMB7 | RPL30 | SLC38A10 | TCP10L | TSEN2 | ZBTB9 |
| ADAP2 | BARD1 | CCNB2 | CYP4F22 | ERG | GHSR | IPO5 | LRP3 | MUC4 | PAX9 | PSMC2 | RPL34 | SLC38A11 | TCTE3 | TSEN54 | ZC3H10 |
| ADD1 | BASP1 | CCNE1 | CYSLTR1 | ERGIC1 | GIMAP1 | IQGAP2 | LRP5 | MUM1L1 | PBOV1 | PSPC1 | RPL35 | SLC38A5 | TCTEX1D2 | TSHZ1 | ZC3H12B |
| ADNP | BBS1 | CCNI | CYTH2 | ERGIC2 | GIMAP7 | IQGAP3 | LRPPRC | MUSK | PCDH10 | PSPH | RPL35A | SLC38A7 | TCTN2 | TSNARE1 | ZC3H13 |
| ADPGK | BBS5 | CCNJ | CYTH3 | ERP27 | GJA5 | IQSEC2 | LRRC28 | MYBL1 | PCDH17 | PSTK | RPL37 | SLC38A9 | TDRG1 | TSPAN11 | ZC3H4 |
| ADPRH | BCDIN3D | CCNL2 | DAAM1 | ESF1 | GJC1 | IRAK1BP1 | LRRC46 | MYEOV2 | PCDH7 | PTAFR | RPL6 | SLC39A13 | TEAD1 | TSPAN18 | ZC3H6 |
| ADPRHL2 | BCL11B | CCNT1 | DAB2IP | ESRRB | GLG1 | IREB2 | LRRC57 | MYLIP | PCDHAC2 | PTAR1 | RPL7L1 | SLC40A1 | TECTA | TSPYL6 | ZC3H8 |
| ADSS | BCL2L12 | CCT5 | DACH2 | ETNK1 | GLI3 | IRF2BP2 | LRRC8A | MYNN | PCDHB14 | PTCD3 | RPLP1 | SLC41A3 | TEP1 | TTC14 | ZC3HC1 |
| AFAP1L2 | BCL2L13 | CD109 | DAPK3 | EVX1 | GLIPR1L1 | IRS1 | LRRC8E | MYO1C | PCDHB3 | PTGR2 | RPP14 | SLC44A1 | TET1 | TTC18 | ZC4H2 |
| AGAP2 | BCL2L14 | CD276 | DAZAP2 | EXD3 | GLIS3 | IRX3 | LRRFIP1 | MYO1G | PCID2 | PTPLAD2 | RPRD2 | SLC45A1 | TEX13A | TTC26 | ZCCHC17 |
| AGAP6 | BCLAF1 | CD47 | DBF4B | EXOC3 | GLS | ISL2 | LRRFIP2 | MYO5B | PCMTD2 | PTPLB | RPS19 | SLC45A4 | TEX264 | TTC27 | ZCCHC2 |
| AGBL5 | BCO2 | CD48 | DCAF16 | EXOC4 | GLTP | ISY1 | LSAMP | MYPOP | PCNT | PTPN1 | RPS23 | SLC46A1 | TFAM | TTC30B | ZCCHC6 |
| AGMAT | BCORL1 | CD5 | DCAF7 | EXOC6B | GLYAT | ISYNA1 | LSG1 | N4BP2 | PCNX | PTPN21 | RPS25 | SLC6A17 | TFB1M | TTC5 | ZCCHC7 |
| AGPAT6 | BCR | CD53 | DCAKD | EXOC8 | GLYCTK | ITFG1 | LSM10 | N6AMT2 | PCP2 | PTPN4 | RPS27A | SLC6A2 | TFDP1 | TTL | ZCCHC9 |
| AGR2 | BDNF | CD84 | DCBLD1 | EXOSC1 | GMCL1 | ITFG3 | LSM11 | NAALADL1 | PCSK7 | PTPN5 | RPS29 | SLC7A3 | TFDP2 | TTPAL | ZDHHC1 |
| AGR3 | BEND3 | CDADC1 | DCBLD2 | EXOSC6 | GMDS | ITGA2 | LSM3 | NADSYN1 | PCYT1A | PTPRS | RPS6KA3 | SLC7A6OS | TFEC | TTTY11 | ZDHHC13 |
| AHCTF1 | BEX2 | CDC23 | DCP1B | EYS | GMIP | ITGB1BP1 | LTV1 | NAGS | PDCD7 | PURA | RPS6KA4 | SLC9A1 | TFRC | TTTY5 | ZDHHC14 |
| AHI1 | BEX5 | CDC34 | DCP2 | F11R | GNAI1 | ITGB2 | LUC7L | NAIF1 | PDE12 | PURB | RPS6KB1 | SLC9A5 | TGFBR3 | TTYH2 | ZDHHC22 |
| AHSA2 | BHMT2 | CDC42 | DCTN2 | F7 | GNAS | ITIH4 | LUC7L2 | NAIP | PDE4A | PURG | RPS7 | SLC9A9 | TGFBRAP1 | TUBD1 | ZDHHC23 |
| AIDA | BIRC3 | CDC42EP5 | DCTN5 | FAAH2 | GNG12 | ITPRIP | LY9 | NANS | PDE6D | PVRL1 | RPUSD1 | SLCO3A1 | THAP6 | TUBGCP5 | ZDHHC4 |
| AKAP10 | BIRC6 | CDC42SE1 | DCUN1D3 | FADS2 | GNG7 | JAGN1 | LYNX1 | NAP1L4 | PDE7B | PVRL2 | RPUSD4 | SLFN11 | THAP9 | TUBGCP6 | ZDHHC5 |
| AKAP13 | BMP8B | CDCA3 | DDI1 | FAF1 | GNPNAT1 | JAK3 | LYPD4 | NAPB | PDE8B | PXMP4 | RRAGC | SLFN5 | THBD | TUFM | ZER1 |
| AKAP5 | BMS1P5 | CDCA5 | DDIT4L | FAHD2A | GOLGA2 | JAM2 | LYPD6 | NAPG | PDIK1L | PYGO2 | RRBP1 | SLMAP | THEM4 | TUG1 | ZFAND2A |
| AKAP8L | BNC2 | CDH11 | DDR2 | FAM101B | GOLGA7B | JAM3 | LYRM5 | NARF | PDK1 | QRICH1 | RREB1 | SMAD1 | THNSL2 | TUSC5 | ZFAND2B |
| AKIRIN1 | BNIP2 | CDH24 | DDX20 | FAM102A | GOPC | JARID2 | LYRM7 | NARS | PDPN | QSER1 | RRM2B | SMAD9 | THOC2 | TXNDC12 | ZFHX3 |
| AKIRIN2 | BOC | CDK9 | DDX31 | FAM103A1 | GORASP1 | JAZF1 | LYSMD2 | NAV1 | PDRG1 | QSOX2 | RRN3P2 | SMARCE1 | THRAP3 | TXNDC15 | ZFP62 |
| AKT3 | BOLA2 | CDKL3 | DDX46 | FAM104A | GPD2 | JDP2 | LZTFL1 | NBAS | PDS5B | RAB10 | RSAD2 | SMC6 | THSD7B | TXNDC16 | ZFP82 |
| AKTIP | BOLA3 | CDKN2B | DDX49 | FAM109A | GPHA2 | JMJD1C | MACF1 | NCAM1 | PDXK | RAB11A | RSBN1 | SMNDC1 | THYN1 | TXNDC17 | ZFYVE1 |
| ALDH18A1 | BOLL | CDS1 | DDX5 | FAM110A | GPIHBP1 | JMY | MAD2L1 | NCDN | PDZD11 | RAB12 | RSF1 | SMU1 | TIGD5 | U2AF1L4 | ZFYVE20 |
| ALDOA | BRD4 | CDYL2 | DDX51 | FAM110B | GPR113 | JOSD2 | MAD2L2 | NCK1 | PEAR1 | RAB1A | RSL24D1 | SMUG1 | TIGD7 | U2AF2 | ZFYVE21 |
| ALG13 | BRD7 | CEBPG | DDX54 | FAM114A1 | GPR155 | JPH2 | MAFB | NCOA5 | PECR | RAB23 | RSPH3 | SMURF2 | TIGIT | UBA2 | ZFYVE27 |
| ALG5 | BREA2 | CENPBD1 | DDX55 | FAM118A | GPR158 | JTB | MAFG | NCOA7 | PELI1 | RAB27B | RSPRY1 | SMYD4 | TIMM23 | UBA5 | ZFYVE28 |
| ALG9 | BRI3 | CENPJ | DDX56 | FAM120B | GPR180 | KALRN | MAGI1 | NCSTN | PELI3 | RAB2B | RTDR1 | SNAP29 | TIMP2 | UBA6 | ZHX1 |
| ALKBH2 | BRPF3 | CENPK | DDX6 | FAM126A | GPR39 | KARS | MAGOHB | NDNL2 | PES1 | RAB30 | RTKN | SNAPIN | TINAG | UBE2B | ZIC1 |
| ALKBH5 | BSND | CENPL | DEDD2 | FAM149B1 | GPR68 | KATNB1 | MAN2A1 | NDRG3 | PEX11G | RAB3GAP1 | RUFY3 | SNIP1 | TINF2 | UBE2E2 | ZIC4 |
| ALKBH6 | BTD | CENPN | DEF8 | FAM159A | GPR83 | KBTBD2 | MAN2C1 | NDUFA11 | PEX26 | RAB43 | RUNX2 | SNRNP200 | TLK2 | UBE2G1 | ZKSCAN1 |
| ALS2 | BUB3 | CEP250 | DEFB123 | FAM160A2 | GPRIN3 | KBTBD4 | MANEAL | NDUFA2 | PEX5L | RAB7A | RWDD2A | SNRPA1 | TLN2 | UBE2G2 | ZKSCAN2 |
| AMDHD1 | BUD13 | CEPT1 | DEFB127 | FAM160B1 | GPSM1 | KCNA7 | MAP1LC3A | NDUFAF2 | PFKFB1 | RAB8B | RYBP | SNTN | TLR7 | UBE2K | ZKSCAN3 |
| AMIGO1 | C10orf11 | CFC1 | DENND1B | FAM160B2 | GPX2 | KCNH3 | MAP2K7 | NDUFB10 | PFKP | RABL3 | S100A10 | SNX12 | TLR9 | UBE2Q1 | ZMAT2 |
| AMMECR1 | C10orf35 | CFLAR | DENND4C | FAM161B | GPX8 | KCNJ10 | MAP3K6 | NDUFB2 | PGAP1 | RAD52 | S100A16 | SNX14 | TM2D2 | UBE2Q2 | ZMAT3 |
| AMMECR1L | C10orf88 | CGA | DEPDC7 | FAM162A | GRAMD1A | KCNJ15 | MAP3K8 | NDUFB4 | PGBD1 | RAD54L2 | S1PR2 | SNX16 | TM6SF2 | UBE2U | ZMIZ1 |
| ANAPC4 | C11orf54 | CHCHD10 | DERL2 | FAM166B | GRB2 | KCNK16 | MAP6D1 | NDUFB9 | PGBD4 | RAI1 | SAAL1 | SNX2 | TM7SF3 | UBE2Z | ZMYM6 |
| ANAPC5 | C11orf57 | CHCHD3 | DFNB59 | FAM167A | GRIN2A | KCNK3 | MAPK1 | NDUFC1 | PGK1 | RALA | SAE1 | SNX22 | TMBIM4 | UBFD1 | ZMYND12 |
| ANGPTL4 | C11orf73 | CHCHD5 | DGCR5 | FAM168B | GRIN2B | KCNK7 | MAPK6 | NDUFV1 | PGM2 | RALBP1 | SALL3 | SNX3 | TMCC3 | UBIAD1 | ZNF124 |
| ANK2 | C11orf83 | CHD1 | DGKE | FAM174A | GRIN3A | KCNK9 | MAPK8 | NECAB3 | PGM2L1 | RALGAPA1 | SALL4 | SNX5 | TMED4 | UBLCP1 | ZNF148 |
| ANKFY1 | C11orf86 | CHD1L | DGKH | FAM175A | GRIPAP1 | KCNQ4 | MARCKS | NECAP2 | PGM5 | RALGAPB | SAMD10 | SOAT1 | TMEM106B | UBQLN2 | ZNF18 |
| ANKIB1 | C12orf29 | CHD2 | DGKK | FAM187B | GRPEL2 | KCTD1 | MAS1L | NEFM | PGS1 | RALGPS1 | SAMD8 | SOCS3 | TMEM119 | UBQLNL | ZNF207 |
| ANKRD10 | C12orf49 | CHID1 | DGKZ | FAM195A | GSG1 | KCTD10 | MBD6 | NES | PHACTR1 | RANBP17 | SAMD9L | SOCS4 | TMEM120A | UBR4 | ZNF213 |
| ANKRD13A | C12orf57 | CHMP4B | DHDDS | FAM24A | GSK3B | KCTD18 | MBNL1 | NETO2 | PHAX | RANBP9 | SAMM50 | SOCS6 | TMEM128 | UBTF | ZNF221 |
| ANKRD16 | C12orf76 | CHMP4C | DHRS13 | FAM24B | GSX1 | KCTD5 | MBOAT1 | NFASC | PHF11 | RAP1GDS1 | SAP30L | SON | TMEM129 | UBXN2A | ZNF233 |
| ANKRD22 | C14orf28 | CHORDC1 | DHRS7 | FAM26E | GTF2H5 | KDM1B | MBP | NFAT5 | PHF12 | RAP2B | SARM1 | SORBS1 | TMEM134 | UCHL5 | ZNF25 |
| ANKRD23 | C15orf41 | CHST14 | DHX33 | FAM26F | GTF3A | KDM2B | MBTD1 | NFATC2 | PHF13 | RAPGEF2 | SARS | SORL1 | TMEM138 | UCKL1 | ZNF253 |
| ANKRD24 | C15orf57 | CHTF8 | DHX37 | FAM43A | GTF3C3 | KDM5B | MCC | NFIB | PHF23 | RAPGEF6 | SASS6 | SP1 | TMEM141 | UEVLD | ZNF254 |
| ANKRD28 | C16orf13 | CIAO1 | DHX57 | FAM47C | GTF3C4 | KGFLP2 | MCEE | NFXL1 | PHF5A | RASAL3 | SAT2 | SP100 | TMEM143 | UFM1 | ZNF260 |
| ANKRD35 | C16orf70 | CIRH1A | DIP2A | FAM58A | GTPBP1 | KHDRBS3 | MCHR1 | NGRN | PHIP | RASGEF1C | SATB1 | SP8 | TMEM14B | UFSP1 | ZNF264 |
| ANKRD40 | C16orf72 | CISD2 | DIS3 | FAM63A | GTPBP8 | KHSRP | MCHR2 | NHLRC3 | PHLDA2 | RASGRP3 | SBNO1 | SPACA3 | TMEM14C | UHMK1 | ZNF271 |
| ANKRD52 | C16orf87 | CISD3 | DIS3L2 | FAM63B | GTSE1 | KIAA0141 | MCL1 | NIP7 | PHRF1 | RASIP1 | SCAMP2 | SPAG16 | TMEM168 | UHRF2 | ZNF276 |
| ANKRD53 | C16orf89 | CKAP2L | DISP1 | FAM76A | GUCA1C | KIAA0907 | MCM6 | NIPA1 | PIGK | RASL11B | SCAMP4 | SPAG7 | TMEM169 | ULK3 | ZNF280B |
| ANKRD54 | C17orf100 | CKAP4 | DISP2 | FAM76B | GUCY1A3 | KIAA1147 | MCM8 | NIPSNAP3A | PIGM | RASSF3 | SCARA5 | SPAG9 | TMEM170A | UNC13D | ZNF281 |
| ANKS4B | C17orf70 | CLDN12 | DKK3 | FAM81B | GUSB | KIAA1161 | MCOLN2 | NISCH | PIK3AP1 | RASSF8 | SCARB2 | SPATA19 | TMEM170B | UNC5B | ZNF302 |
| ANKS6 | C17orf80 | CLDN18 | DLC1 | FAM83F | H6PD | KIAA1244 | MCTS1 | NKAPL | PIK3C3 | RASSF9 | SCARNA15 | SPATA5 | TMEM171 | UNC5C | ZNF317 |
| ANLN | C17orf85 | CLDN23 | DLG1 | FAM84A | HACE1 | KIAA1324L | MDM4 | NKD2 | PIK3CG | RAVER1 | SCARNA17 | SPATA7 | TMEM173 | UNK | ZNF319 |
| ANO6 | C17orf89 | CLDND1 | DLL3 | FAM91A1 | HAGHL | KIAA1328 | MED10 | NLGN3 | PIK3R5 | RAVER2 | SCFD1 | SPATS2 | TMEM176B | UQCRC2 | ZNF324B |
| ANO9 | C17orf96 | CLDND2 | DLL4 | FAM96A | HAS3 | KIAA1522 | MED13L | NLK | PIM3 | RBM12B | SCFD2 | SPEM1 | TMEM18 | URM1 | ZNF326 |
| ANXA1 | C18orf21 | CLEC14A | DLX1 | FAM98A | HBS1L | KIAA1586 | MED23 | NNMT | PIP5KL1 | RBM15 | SCGB3A2 | SPG20 | TMEM192 | USP16 | ZNF33A |
| AP1G1 | C18orf25 | CLEC4E | DMRT2 | FAM98B | HCFC2 | KIAA1755 | MED31 | NOB1 | PITRM1 | RBM15B | SCN7A | SPIN1 | TMEM194B | USP3 | ZNF341 |
| AP4B1 | C19orf25 | CLINT1 | DMXL1 | FAM98C | HDAC11 | KIAA2013 | MED7 | NOBOX | PLA2G16 | RBM17 | SCOC | SPNS1 | TMEM200B | USP30 | ZNF346 |
| APBB1IP | C19orf26 | CLIP3 | DNAH9 | FANCA | HDAC4 | KIAA2018 | MEF2D | NOL10 | PLAC4 | RBM22 | SCRT1 | SPNS2 | TMEM203 | USP31 | ZNF347 |
| APEH | C19orf44 | CLNS1A | DNAJA2 | FANCC | HDAC7 | KIF19 | MEGF10 | NONO | PLAC9 | RBM33 | SCYL2 | SPOP | TMEM206 | USP37 | ZNF354C |
| APEX2 | C1GALT1 | CLOCK | DNAJB11 | FANCD2 | HDHD2 | KIF1B | MEGF8 | NOSTRIN | PLCG2 | RBM39 | SDF4 | SPRED3 | TMEM207 | USP38 | ZNF358 |
| APIP | C1orf122 | CLPTM1L | DNAJC1 | FANCM | HEATR1 | KIF23 | MEIG1 | NOTUM | PLCXD1 | RBM41 | SDHAF2 | SPRN | TMEM216 | USP40 | ZNF362 |
| APOA1BP | C1orf131 | CLPX | DNAJC18 | FANK1 | HECTD1 | KIF26A | MESDC2 | NOVA2 | PLCXD2 | RBM43 | SDR16C5 | SPRYD4 | TMEM218 | USP42 | ZNF395 |
| APOA5 | C1orf158 | CLSPN | DNAJC21 | FBLN7 | HECW1 | KIF26B | METRNL | NOXA1 | PLEKHA7 | RBM45 | SDR42E1 | SPSB4 | TMEM219 | USP47 | ZNF404 |
| APTX | C1orf162 | CLUAP1 | DNAJC27 | FBRS | HELZ | KIFC2 | METTL14 | NOXO1 | PLEKHA8 | RBM47 | SEC11C | SQSTM1 | TMEM229B | USP7 | ZNF414 |
| ARAP1 | C1orf192 | CLYBL | DNAJC3 | FBXL12 | HEPACAM2 | KITLG | METTL3 | NPFFR2 | PLEKHB2 | RBM5 | SEC16B | SRCRB4D | TMEM33 | USP9X | ZNF416 |
| ARF1 | C1orf43 | CMBL | DNAJC30 | FBXL19 | HERPUD2 | KLB | METTL7A | NPM1 | PLEKHG4 | RBM6 | SEC62 | SRGAP1 | TMEM41A | UTP11L | ZNF425 |
| ARF3 | C1orf87 | CMIP | DNAJC9 | FBXL6 | HES4 | KLF12 | METTL7B | NPNT | PLEKHG5 | RBM8A | SEC63 | SRP68 | TMEM42 | UTP23 | ZNF431 |
| ARFGEF2 | C1orf95 | CMKLR1 | DNHD1 | FBXO15 | HEXA | KLF13 | MEX3A | NPW | PLG | RBMS2 | SECISBP2 | SRRD | TMEM43 | UTP6 | ZNF436 |
| ARGLU1 | C1QTNF7 | CMTM5 | DNMT3A | FBXO3 | HEXIM2 | KLF3 | MEX3B | NR2C1 | PLRG1 | RBP1 | SELO | SRXN1 | TMEM44 | UXS1 | ZNF439 |
| ARHGAP1 | C20orf144 | CNDP2 | DNTTIP1 | FBXO31 | HHLA1 | KLF7 | MEX3C | NR2C2AP | PLSCR2 | RBP2 | SEMA4A | SSBP4 | TMEM50B | VANGL1 | ZNF451 |
| ARHGAP21 | C21orf59 | CNKSR2 | DOCK1 | FBXO33 | HIAT1 | KLF9 | MEX3D | NR2F2 | PLXDC1 | RBPMS | SEMA4F | SSFA2 | TMEM54 | VASH1 | ZNF462 |
| ARHGAP30 | C22orf34 | CNOT3 | DOCK11 | FBXO38 | HIATL1 | KLHDC8B | MFI2 | NRAP | PLXNA2 | RCCD1 | SEMA6B | SSR3 | TMEM55A | VASN | ZNF470 |
| ARHGAP5 | C22orf39 | CNOT6L | DOCK8 | FBXW8 | HIBADH | KLHL14 | MFSD4 | NRK | PM20D1 | RCHY1 | SEMA6D | SSTR1 | TMEM56 | VAV2 | ZNF507 |
| ARHGEF12 | C2CD3 | CNPY3 | DOK3 | FCHO2 | HIGD1A | KLHL15 | MFSD6 | NRM | PM20D2 | RCOR3 | SEMA7A | ST3GAL2 | TMEM59 | VGLL2 | ZNF512 |
| ARID1B | C2orf49 | COG3 | DPP3 | FCRL1 | HINT1 | KLHL24 | MGAT4A | NRXN1 | PMCHL1 | RD3 | SENP8 | ST8SIA3 | TMEM60 | VPS13A | ZNF521 |
| ARID2 | C2orf57 | COG5 | DPP7 | FCRLA | HIPK2 | KLHL36 | MGAT4B | NRXN3 | PML | RDH13 | SEPSECS | STAB2 | TMEM61 | VPS18 | ZNF529 |
| ARID4A | C2orf68 | COG8 | DPP9 | FDX1L | HIRIP3 | KLHL5 | MGAT5B | NSMCE1 | PMS1 | RDM1 | SERGEF | STAC3 | TMEM62 | VPS25 | ZNF532 |
| ARIH2 | C2orf70 | COL22A1 | DPYSL3 | FER | HIST1H4H | KPNA4 | MGEA5 | NSMCE2 | PNKD | RDX | SERP2 | STAMBPL1 | TMEM68 | VPS26A | ZNF536 |
| ARL16 | C3orf67 | COL4A3BP | DPYSL5 | FFAR1 | HIVEP2 | KPNA5 | MICAL2 | NSMCE4A | PNPLA5 | REEP3 | SESN3 | STK10 | TMEM70 | VPS26B | ZNF544 |
| ARL8A | C4orf29 | COL4A4 | DSCAM | FGA | HM13 | KPNA6 | MICALL2 | NSUN2 | PNPLA8 | RELT | SETD8 | STK11 | TMEM71 | VPS33A | ZNF551 |
| ARL8B | C4orf47 | COL9A2 | DSEL | FGF18 | HMBOX1 | KRBA1 | MIER1 | NSUN3 | PNPO | REM2 | SETDB2 | STK11IP | TMEM87A | VPS35 | ZNF557 |
| ARMC5 | C5orf15 | COMMD10 | DST | FGF19 | HMGCLL1 | KREMEN1 | MIER3 | NSUN6 | PNRC2 | RETNLB | SEZ6L2 | STK16 | TMEM8B | VPS41 | ZNF559 |
| ARMC8 | C5orf34 | COMMD7 | DTD1 | FGFR1 | HMX2 | KRI1 | MIF4GD | NTNG2 | POGK | REV1 | SFMBT2 | STK36 | TMEM9 | VPS8 | ZNF566 |
| ARMC9 | C5orf56 | COMTD1 | DTL | FGFRL1 | HN1 | KRIT1 | MINA | NUB1 | POLB | REV3L | SFPQ | STK4 | TMEM91 | VSIG2 | ZNF581 |
| ARPC5L | C6orf136 | COPB2 | DTWD2 | FIZ1 | HNF4A | KRT73 | MITD1 | NUCKS1 | POLDIP3 | REXO1 | SFT2D1 | STMN3 | TMEM99 | VSTM1 | ZNF582 |
| ARRDC1 | C6orf203 | COPS4 | DUOXA2 | FKBP5 | HNF4G | L2HGDH | MKLN1 | NUDCD1 | POLE4 | REXO2 | SGCB | STOX2 | TMEM9B | WASF2 | ZNF598 |
| ARRDC3 | C8orf76 | COPS7B | DUSP15 | FLCN | HNRNPA2B1 | L3MBTL4 | MLLT4 | NUDCD3 | POLH | RFX1 | SH3KBP1 | STRADB | TMF1 | WBP11 | ZNF606 |
| ARRDC4 | C9orf117 | COPZ2 | DUSP18 | FLI1 | HNRNPC | LACTB | MLLT6 | NUDT14 | POLN | RFX4 | SH3RF1 | STRBP | TMPRSS13 | WBSCR22 | ZNF608 |
| ARSA | C9orf40 | COQ2 | DUSP27 | FLJ16779 | HNRNPK | LAMB4 | MLYCD | NUDT16 | POLR2F | RFX7 | SH3TC2 | STRN | TMTC1 | WDR13 | ZNF615 |
| ARSG | CA10 | COQ5 | DUSP28 | FLJ40288 | HNRNPR | LAMP2 | MMAA | NUFIP2 | POLR3E | RGL3 | SHF | STT3B | TMUB1 | WDR19 | ZNF616 |
| ARX | CAB39 | CORO1C | DYM | FLJ42627 | HNRNPUL2 | LARP4B | MMAB | NUMA1 | POLR3K | RGS12 | SHISA4 | STX3 | TMX3 | WDR24 | ZNF621 |
| ASAP1 | CAB39L | COTL1 | DYNC1LI1 | FLVCR1 | HOMEZ | LARP6 | MMP15 | NUMB | POMGNT1 | RGS18 | SHISA5 | STX6 | TNFAIP8L2 | WDR27 | ZNF628 |
| ASB1 | CABLES2 | COX15 | DYNLL2 | FLVCR2 | HORMAD1 | LARP7 | MMRN2 | NUMBL | POMP | RGS5 | SHKBP1 | STX7 | TNFRSF10C | WDR34 | ZNF641 |
| ASB12 | CACNA1C | COX18 | DYRK1A | FLYWCH1 | HOXA3 | LARS | MOGAT2 | NUP133 | POMT2 | RHBDD1 | SHPRH | STYX | TNFRSF11A | WDR36 | ZNF649 |
| ASCC3 | CACNA1D | COX19 | DZIP1L | FNBP1 | HOXB1 | LAS1L | MON1A | NUP35 | PON2 | RHBDD2 | SHROOM1 | SUB1 | TNIP1 | WDR37 | ZNF653 |
| ASCL2 | CACNG4 | COX5A | EAF1 | FNDC7 | HP1BP3 | LCLAT1 | MON2 | NUP37 | POU2F1 | RHEB | SIK2 | SUCNR1 | TNIP2 | WDR41 | ZNF655 |
| ASH1L | CALCOCO2 | COX7B | EBF1 | FNIP2 | HS2ST1 | LCN10 | MORN1 | NUP54 | POU6F1 | RHOA | SIN3A | SUDS3 | TNPO2 | WDR54 | ZNF664 |
| ASNS | CALD1 | CPD | ECHDC1 | FOLR2 | HSD17B1 | LCP2 | MOXD1 | NUP93 | PPAPDC1B | RHOT1 | SIN3B | SUFU | TNRC6A | WDR55 | ZNF667 |
| ASPRV1 | CAMK2G | CPLX2 | EDEM3 | FOSL2 | HSF5 | LDB2 | MPRIP | NXPH1 | PPDPF | RIC8B | SIRPG | SULT1E1 | TNS3 | WDR59 | ZNF668 |
| ASXL1 | CAMKK1 | CPM | EDN1 | FOXA3 | HSPA14 | LDB3 | MPV17L2 | OAF | PPIE | RICTOR | SIRT6 | SUMF1 | TOM1L2 | WDR6 | ZNF672 |

**Table S5. Prognostic analysis of 20 genes used to construct the m6A scoring model**

| **Gene** | **p Value** | **HR** | **Low 95%CI** | **High 95%CI** |
| --- | --- | --- | --- | --- |
| CAB39 | 0.001 | 1.77 | 1.25 | 2.51 |
| DCTN5 | 0.001 | 1.78 | 1.25 | 2.52 |
| SPIN1 | 0.003 | 1.7 | 1.2 | 2.4 |
| L2HGDH | 0.006 | 0.61 | 0.43 | 0.87 |
| ARAP1 | 0.007 | 1.61 | 1.14 | 2.28 |
| ANKRD16 | 0.009 | 0.63 | 0.45 | 0.89 |
| MCM6 | 0.01 | 0.63 | 0.45 | 0.9 |
| ZNF830 | 0.011 | 1.57 | 1.11 | 2.22 |
| MCL1 | 0.012 | 1.56 | 1.1 | 2.21 |
| PCNT | 0.018 | 0.65 | 0.46 | 0.93 |
| MRPL14 | 0.02 | 0.67 | 0.47 | 0.94 |
| SNX2 | 0.023 | 0.67 | 0.47 | 0.94 |
| SCFD1 | 0.024 | 1.49 | 1.05 | 2.11 |
| NDUFAF2 | 0.036 | 0.69 | 0.49 | 0.98 |
| MIF4GD | 0.038 | 0.69 | 0.49 | 0.98 |
| RNF219 | 0.038 | 1.44 | 1.02 | 2.04 |
| ZNF436 | 0.043 | 1.43 | 1.01 | 2.02 |
| DDX54 | 0.048 | 0.71 | 0.5 | 1 |
| MAPK8 | 0.048 | 1.42 | 1 | 2 |
| TMEM203 | 0.049 | 0.71 | 0.5 | 1 |

**Table S6. Multivariate Cox regression analysis of m6A score**

| **Characteristics** | **HR(95%CI)** | P value |
| --- | --- | --- |
| Stage |  |  |
| I-II | Reference |  |
| III-IV | 1.632(0.763-3.489) | 0.206 |
| Number of Extra nodal sites |  |  |
| ≤1 | Reference |  |
| >1 | 1.198(0.488-2.941) | 0.693 |
| Age |  |  |
| ≤60 years | Reference |  |
| >60 years | 1.457(0.752-2.824) | 0.265 |
| LDH ratio |  |  |
| ≤1 | Reference |  |
| >1 | 1.177(0.633-2.188) | 0.607 |
| IPI score |  |  |
| 1 | Reference |  |
| 2 | 1.787(0.818-3.906) | 0.145 |
| 3 | 1.899(0.359-10.042) | 0.451 |
| m6A score |  |  |
| high score | Reference |  |
| low score | 2.757(1.338-5.682) | 0.006 |

**Table S7. Clinical information of patients in the IMvigor210 cohort receiving anti-PD-L1 therapy**

| **Sample ID** | **Neoantigen burden per MB** | **PD-L1 expressioin on tumor cells *** | **Clinical Response** | **Time (months)** | **Status** | **m6A score** | **m6A score group** | **TIS** | **TIDE** |
| --- | --- | --- | --- | --- | --- | --- | --- | --- | --- |
| SAM0257bbbbd388 | 4.68627451 | TC0 | SD/PD | 15.6386037 | dead | -1.34813635 | low | 2.415877518 | -1.07 |
| SAM025b45c27e05 | 0.31372549 | TC0 | SD/PD | 8.772073922 | dead | 2.565322732 | high | 2.181906942 | 2.53 |
| SAM032c642382a7 | 6.196078431 | TC2+ | SD/PD | 2.496919918 | dead | -0.089550248 | high | 3.146576307 | -1.14 |
| SAM0571f17f4045 | 1.470588235 | TC0 | SD/PD | 4.533880903 | dead | -0.399986193 | high | 3.173892511 | 0.87 |
| SAM065890737112 | NA | TC0 | CR/PR | 20.07392197 | alive | -1.465501161 | low | 2.793969526 | -1.42 |
| SAM0684af734db1 | 0.921568627 | TC0 | CR/PR | 23.26078029 | alive | 0.343100666 | high | 2.220292063 | -0.22 |
| SAM075e037d95bc | 0.745098039 | TC0 | CR/PR | 21.32238193 | alive | -0.874002927 | low | 2.241711741 | -0.92 |
| SAM07a93a28f801 | 2.117647059 | TC0 | CR/PR | 12.84599589 | alive | -3.961283073 | low | 2.241940428 | 0.19 |
| SAM08cce2fa88f2 | NA | TC0 | SD/PD | 22.11088296 | alive | 2.098498101 | high | 1.941981131 | -0.62 |
| SAM09c84ec0cf34 | NA | TC0 | SD/PD | 6.50513347 | dead | 1.505533944 | high | 1.706257155 | 1.31 |
| SAM0a0f2bac4b20 | 0.215686275 | TC1 | SD/PD | 1.18275154 | dead | 4.29606706 | high | 2.641428673 | 0.45 |
| SAM0a7c2091dd56 | 1.274509804 | TC1 | SD/PD | 2.628336756 | dead | 3.089488272 | high | 2.681571474 | 0.95 |
| SAM0bdb3428bd13 | NA | TC0 | SD/PD | 2.234086242 | dead | 0.377498579 | high | 2.367506232 | 2.04 |
| SAM0ce9c983b20f | NA | TC0 | SD/PD | 6.603696099 | dead | 0.300168789 | high | 2.709999985 | 0.4 |
| SAM0d855cff64e6 | 0.137254902 | TC0 | SD/PD | 2.858316222 | dead | 0.971008475 | high | 2.324290599 | -0.39 |
| SAM0f956e757453 | 4.098039216 | TC0 | SD/PD | 10.41478439 | dead | -0.257517133 | high | 2.440859228 | 1.11 |
| SAM110501d0eedb | 3.529411765 | TC0 | SD/PD | 4.271047228 | dead | -3.052553797 | low | 2.596146011 | -0.82 |
| SAM12502d970c10 | 1.078431373 | TC0 | SD/PD | 7.392197125 | dead | 0.634135099 | high | 2.33407311 | -0.22 |
| SAM14938611a2d3 | 6.68627451 | TC0 | CR/PR | 21.42094456 | alive | -1.729636268 | low | 2.604283998 | -1.07 |
| SAM14df63a65411 | 0.568627451 | TC2+ | CR/PR | 21.42094456 | alive | 1.602774176 | high | 2.46050022 | -0.36 |
| SAM166a419a4e5a | 0.274509804 | TC0 | SD/PD | 5.388090349 | dead | 4.323946284 | high | 2.69135821 | 2.46 |
| SAM18039827e1b9 | NA | TC0 | SD/PD | 1.478439425 | dead | -0.703624998 | high | 2.172790994 | -0.71 |
| SAM181b638b8248 | 0.215686275 | TC2+ | SD/PD | 10.87474333 | dead | 4.866919755 | high | 2.89894562 | 0.2 |
| SAM187e056d6a2a | NA | TC0 | SD/PD | 2.299794661 | dead | -0.262266464 | high | 2.207679263 | 1.13 |
| SAM18a4dabbc557 | 2.039215686 | TC2+ | SD/PD | 16.45995893 | dead | -1.023617422 | low | 2.769639004 | -1.31 |
| SAM18b9351e265a | 0.392156863 | TC0 | SD/PD | 15.86858316 | dead | -0.796945102 | high | 2.971097456 | -0.17 |
| SAM18bc1078bc15 | 1.803921569 | TC0 | SD/PD | 6.899383984 | dead | -0.329466829 | high | 2.682237437 | -0.74 |
| SAM18be5b395318 | 0.470588235 | TC1 | SD/PD | 9.002053388 | dead | 0.916214614 | high | 2.594019892 | -1.05 |
| SAM19fec8f3b3bd | 0.431372549 | TC2+ | SD/PD | 9.889117043 | dead | 3.738274403 | high | 2.390217279 | 1.25 |
| SAM1a87df750b9d | 1.176470588 | TC0 | SD/PD | 5.388090349 | dead | -1.970657437 | low | 2.765041167 | -0.2 |
| SAM1ab1b28d9f2b | 0.294117647 | TC0 | SD/PD | 2.102669405 | dead | -4.450947523 | low | 2.234687779 | 0.05 |
| SAM1abf01dd4544 | 1.862745098 | TC0 | CR/PR | 16.85420945 | alive | -0.501884923 | high | 2.603859262 | -0.42 |
| SAM1ac4e3dee297 | 0.647058824 | TC0 | SD/PD | 5.519507187 | dead | -1.157656038 | low | 1.970949349 | -0.6 |
| SAM1c0ecfb3eb63 | NA | TC0 | SD/PD | 5.848049281 | dead | 2.235086895 | high | 2.937240237 | 1.02 |
| SAM1c8b086175ca | 0.549019608 | TC0 | SD/PD | 20.76386037 | dead | 2.447159445 | high | 2.451666785 | 2.93 |
| SAM1dda30f1c5be | 4.019607843 | TC0 | SD/PD | 21.25667351 | alive | 5.182055872 | high | 2.535292486 | 2.09 |
| SAM1e9c4d1d39ae | 1.431372549 | TC0 | SD/PD | 12.41889117 | dead | -2.861943447 | low | 2.591098764 | -0.95 |
| SAM1f66db567eb5 | 4.509803922 | TC0 | CR/PR | 16.59137577 | alive | -0.612912287 | high | 2.89747604 | 1.13 |
| SAM1f83ebd6be9b | 0.549019608 | TC0 | SD/PD | 3.515400411 | dead | -0.846725458 | high | 2.293364725 | -0.76 |
| SAM1fa6bcb7fc48 | 1.235294118 | TC0 | SD/PD | 22.73511294 | alive | -1.015164741 | low | 2.713314026 | -0.9 |
| SAM203dcf14f927 | 2.392156863 | TC1 | CR/PR | 19.35112936 | alive | 0.436135324 | high | 3.055105202 | 0.09 |
| SAM2070b416069c | 0.294117647 | TC1 | SD/PD | 8.016427105 | dead | -0.453179339 | high | 2.464545981 | -1.3 |
| SAM23095936e611 | 0.235294118 | TC0 | SD/PD | 2.234086242 | dead | -2.264194873 | low | 2.209965743 | 0.13 |
| SAM23aa15d4a0b0 | 0.333333333 | TC0 | SD/PD | 5.716632444 | dead | -1.080247325 | low | 2.281175839 | -0.08 |
| SAM25510f300d79 | 1.823529412 | TC0 | CR/PR | 20.69815195 | alive | -2.570849889 | low | 1.959188204 | 0.7 |
| SAM2570ff4aae6e | 1.529411765 | TC0 | CR/PR | 22.37371663 | alive | 3.831055488 | high | 2.91065046 | 0.15 |
| SAM26104d5adc89 | 0.274509804 | TC0 | SD/PD | 5.388090349 | dead | 1.225478094 | high | 2.085264478 | 1.9 |
| SAM2624229effe8 | 0.431372549 | TC0 | SD/PD | 2.562628337 | dead | 1.763900978 | high | 2.509695076 | 2.63 |
| SAM27299aed7681 | 0.745098039 | TC0 | CR/PR | 18.85831622 | alive | -3.248626502 | low | 2.380808517 | -0.67 |
| SAM28687037e4ff | 0.568627451 | TC2+ | SD/PD | 21.58521561 | alive | 3.695308684 | high | 3.181349914 | 0.4 |
| SAM28e6031ac18b | NA | TC0 | SD/PD | 9.232032854 | dead | 0.511444723 | high | 1.879733373 | -0.68 |
| SAM297c0301e861 | NA | TC2+ | SD/PD | 8.082135524 | dead | 2.693151196 | high | 2.765143092 | 1.31 |
| SAM29da928587ad | 0.490196078 | TC0 | SD/PD | 2.595482546 | dead | -0.709956857 | high | 2.382805473 | 0.9 |
| SAM2b672f4336c7 | 0.156862745 | TC0 | SD/PD | 15.3100616 | dead | 0.134598602 | high | 2.405302007 | -0.36 |
| SAM2bba8cb35e48 | 1.294117647 | TC0 | SD/PD | 10.48049281 | dead | 4.324529845 | high | 2.442377751 | 2.01 |
| SAM2c9586161ce6 | 0.431372549 | TC0 | SD/PD | 11.26899384 | dead | -1.507635345 | low | 2.030160379 | -0.94 |
| SAM2dc3f04e45e9 | NA | TC2+ | SD/PD | 10.18480493 | dead | 0.072317366 | high | 3.00676 | 0.22 |
| SAM2e7aa8fa0ab3 | 2.705882353 | TC0 | SD/PD | 7.72073922 | dead | -0.24585953 | high | 2.662922111 | -0.48 |
| SAM2e9ac0b1b250 | 1.764705882 | TC2+ | CR/PR | 22.14373717 | alive | -1.73108824 | low | 2.957429592 | 0.59 |
| SAM2eb07dedf07f | 1.137254902 | TC0 | CR/PR | 20.50102669 | alive | 2.84857887 | high | 3.14509145 | 0.55 |
| SAM2f228939632f | 0.450980392 | TC0 | SD/PD | 7.885010267 | dead | 1.210734683 | high | 2.695871865 | 0.92 |
| SAM30b5c6c54cf7 | NA | TC0 | CR/PR | 9.264887064 | dead | -4.843793683 | low | 2.58120971 | -0.06 |
| SAM30cf07d4874f | NA | TC0 | SD/PD | 11.66324435 | dead | 3.419333303 | high | 2.631214387 | 0.75 |
| SAM31d9176e11fb | 4.235294118 | TC1 | CR/PR | 17.11704312 | alive | -2.648561977 | low | 2.874074714 | 0.06 |
| SAM31f41dd0d6ca | 0.705882353 | TC0 | SD/PD | 8.27926078 | dead | -0.299261252 | high | 2.640146336 | 0.67 |
| SAM3330c03fdf00 | NA | TC0 | SD/PD | 19.18685832 | alive | 2.008300719 | high | 3.04763107 | 0.93 |
| SAM34430ef08e5b | NA | NA | SD/PD | 18.16837782 | alive | 1.537779535 | high | 2.626610488 | -0.51 |
| SAM36851bc8b9ae | 0.333333333 | TC2+ | SD/PD | 3.54825462 | dead | -2.011970574 | low | 2.462953563 | -0.71 |
| SAM36a9225b0222 | 0.607843137 | TC2+ | SD/PD | 23.81930185 | alive | 3.614715895 | high | 3.145901263 | 0.56 |
| SAM36d87392593b | 0.352941176 | TC2+ | SD/PD | 23.68788501 | alive | -1.755248834 | low | 2.721707312 | -0.14 |
| SAM3894ac3956a5 | NA | TC0 | SD/PD | 2.102669405 | alive | 0.775624015 | high | 2.450332129 | 0.99 |
| SAM39eb94fa504d | NA | TC0 | SD/PD | 17.90554415 | dead | -0.010448918 | high | 2.71705188 | -0.14 |
| SAM3a1c9632ff7b | NA | TC0 | SD/PD | 16.03285421 | alive | -5.538603661 | low | 2.153824982 | 0.22 |
| SAM3b1066e5801b | 0.176470588 | TC0 | SD/PD | 15.6386037 | alive | -0.419560224 | high | 2.896079345 | -1.34 |
| SAM3b15b4c6311d | 5.823529412 | TC2+ | CR/PR | 21.45379877 | alive | 1.393100909 | high | 3.042788625 | 0.05 |
| SAM3cb94b0d5297 | 2.235294118 | TC0 | SD/PD | 5.059548255 | dead | 0.247264709 | high | 2.675305994 | -0.83 |
| SAM3e04eb914f3d | 1.411764706 | TC0 | CR/PR | 17.28131417 | alive | -1.404773375 | low | 2.953760753 | 0.44 |
| SAM3e8baff50d7a | 1.215686275 | TC0 | SD/PD | 6.965092402 | dead | -7.324714387 | low | 2.181842521 | -1.12 |
| SAM3ee5dcd894f0 | NA | TC0 | SD/PD | 8.706365503 | dead | -1.381430214 | low | 2.412880827 | -0.02 |
| SAM415f36ad349e | 0.431372549 | TC0 | SD/PD | 17.97125257 | dead | 2.857223085 | high | 2.433044123 | 0.25 |
| SAM4305ab968b90 | 0.392156863 | TC0 | SD/PD | 1.412731006 | dead | -4.710640027 | low | 1.881901852 | -0.78 |
| SAM4501e41e4751 | 0.921568627 | TC1 | SD/PD | 1.117043121 | dead | 2.909538052 | high | 2.673417924 | 0.28 |
| SAM45c8e6412c66 | 1.392156863 | TC2+ | SD/PD | 3.482546201 | dead | -0.603396023 | high | 3.096276509 | -0.01 |
| SAM468a9e1dc821 | NA | TC0 | SD/PD | 3.876796715 | dead | -2.779769707 | low | 2.079777204 | 0 |
| SAM47fc46c3d6be | 0.803921569 | TC0 | SD/PD | 18.72689938 | alive | -1.329707454 | low | 3.019309149 | 0.44 |
| SAM49d48750e294 | 0.607843137 | TC0 | SD/PD | 9.856262834 | dead | 2.458920815 | high | 2.280819186 | 2.42 |
| SAM49f9b2e57aa5 | 1.450980392 | TC0 | SD/PD | 7.457905544 | dead | -1.857534035 | low | 2.218381598 | -0.42 |
| SAM4b0175e8db6e | NA | TC0 | SD/PD | 2.069815195 | dead | 0.391501981 | high | 2.452457388 | 0.03 |
| SAM4b7ea015fd9e | NA | TC0 | SD/PD | 13.27310062 | dead | 2.595305949 | high | 1.885386904 | -1.01 |
| SAM4caabd64e7fd | 11.68627451 | TC0 | CR/PR | 15.70431211 | alive | -0.357692811 | high | 2.362628451 | -0.55 |
| SAM5234688806a7 | 0.31372549 | TC0 | SD/PD | 5.880903491 | dead | -0.557022077 | high | 2.222747886 | -0.78 |
| SAM52500cabdd36 | 0.745098039 | TC0 | SD/PD | 20.46817248 | alive | -3.206899804 | low | 2.380680462 | -0.29 |
| SAM52e3fa3ad574 | 0.254901961 | TC0 | SD/PD | 7.950718686 | dead | -1.733357741 | low | 3.017935432 | 1.15 |
| SAM548551ef782c | 1.823529412 | TC0 | CR/PR | 17.28131417 | alive | -0.2310953 | high | 2.257031496 | -0.58 |
| SAM54e58f1b0230 | NA | TC0 | SD/PD | 4.369609856 | dead | -0.179599347 | high | 1.962560956 | -0.22 |
| SAM553c3c35b847 | NA | TC0 | SD/PD | 6.702258727 | dead | 3.656532665 | high | 3.237048564 | 0.99 |
| SAM557dde1b9f3e | NA | TC0 | SD/PD | 6.702258727 | dead | -1.795632977 | low | 2.610960809 | 0.59 |
| SAM560f23d6a3ad | 0.647058824 | TC2+ | CR/PR | 21.38809035 | alive | 1.42317662 | high | 3.327011017 | 0.34 |
| SAM563d6233dfa2 | 0.607843137 | TC0 | SD/PD | 2.135523614 | dead | -0.134550395 | high | 1.894098728 | 0.25 |
| SAM568ce160abd9 | NA | TC0 | SD/PD | 12.84599589 | dead | 2.666079999 | high | 2.399875403 | 1.35 |
| SAM572f19794c96 | 5.509803922 | TC2+ | CR/PR | 16.82135524 | alive | -0.269362347 | high | 2.723021432 | -0.16 |
| SAM5767dd75d142 | NA | TC0 | CR/PR | 18.16837782 | alive | -2.383807445 | low | 2.644707694 | -0.57 |
| SAM58e7832f4e7d | 0.333333333 | TC0 | CR/PR | 16.45995893 | alive | -1.793943244 | low | 1.605680549 | -0.88 |
| SAM59f392864f5d | NA | TC0 | SD/PD | 17.77412731 | dead | 4.555376901 | high | 2.79269999 | 0.17 |
| SAM59fda9035d1d | NA | TC0 | SD/PD | 3.745379877 | dead | 2.562632707 | high | 2.205193571 | 3.05 |
| SAM5a2347c0498a | 1.333333333 | TC0 | SD/PD | 4.895277207 | dead | -2.034431939 | low | 2.171013183 | -1.24 |
| SAM5b57e47fdcb3 | NA | TC0 | SD/PD | 3.843942505 | dead | 0.271716311 | high | 2.509368139 | -0.56 |
| SAM5c139c5c1c4f | NA | TC0 | SD/PD | 7.227926078 | dead | 0.733853537 | high | 2.465663049 | -0.56 |
| SAM5cc2d9036053 | 0.411764706 | TC0 | SD/PD | 5.683778234 | dead | -1.225853762 | low | 2.254373427 | 1.58 |
| SAM5cfa1699bdb7 | 1.215686275 | TC0 | SD/PD | 15.83572895 | alive | 0.445584287 | high | 2.607228177 | 0.86 |
| SAM5d1dfd5207f5 | NA | TC0 | SD/PD | 10.90759754 | dead | 0.49595923 | high | 1.897586467 | -0.72 |
| SAM5d989c86255e | 1.411764706 | TC0 | SD/PD | 3.449691992 | dead | 0.544999904 | high | 2.40383671 | 1.35 |
| SAM5e3bae090b8c | 0.823529412 | TC0 | SD/PD | 6.735112936 | dead | 3.111484496 | high | 2.621827276 | -0.79 |
| SAM5fe7a81a39dd | 3.823529412 | TC0 | SD/PD | 17.08418891 | dead | 0.158333119 | high | 2.756466272 | -0.38 |
| SAM6083aac8db99 | NA | TC0 | CR/PR | 20.63244353 | alive | -1.402878191 | low | 2.83556736 | -0.84 |
| SAM6157c8f38b72 | 1.803921569 | TC0 | CR/PR | 16.59137577 | alive | -0.225262568 | high | 2.664948413 | 1.56 |
| SAM61b9d4d84c64 | 5.882352941 | TC0 | SD/PD | 21.38809035 | alive | 0.424154937 | high | 2.631158959 | -1.37 |
| SAM61baf919bb01 | NA | TC0 | SD/PD | 15.60574949 | dead | -1.677239022 | low | 2.874915403 | 1.05 |
| SAM62fb1388c871 | 0.784313725 | TC0 | SD/PD | 22.50513347 | alive | -3.704834631 | low | 2.363565563 | -0.37 |
| SAM63405b04ab2d | 0.156862745 | TC0 | SD/PD | 6.800821355 | dead | 1.731638198 | high | 2.896363701 | 1.84 |
| SAM63b2189c36d7 | 0.980392157 | TC0 | SD/PD | 3.975359343 | dead | 0.092016453 | high | 2.031586207 | -1.01 |
| SAM65afda25b920 | 0.294117647 | TC2+ | SD/PD | 8.082135524 | dead | 2.404764949 | high | 2.840624461 | 0.61 |
| SAM6662f5181f87 | 0.490196078 | TC0 | SD/PD | 19.28542094 | alive | -0.132909587 | high | 2.700962759 | 0.82 |
| SAM670649e105b5 | 2.68627451 | TC0 | CR/PR | 22.07802875 | alive | 0.542867518 | high | 2.658564795 | 0.59 |
| SAM675a12a09c15 | 1.254901961 | TC0 | SD/PD | 2.891170431 | dead | 0.256014523 | high | 2.131391678 | 0.9 |
| SAM6780ed436b55 | 2.549019608 | TC0 | SD/PD | 6.242299795 | dead | -1.490128916 | low | 2.809602056 | -0.05 |
| SAM6792d6e98068 | 1.117647059 | TC0 | SD/PD | 6.012320329 | dead | -1.421329899 | low | 1.98596393 | -0.6 |
| SAM681e4bf7cf85 | 1.862745098 | TC0 | CR/PR | 23.39219713 | alive | -0.781916423 | high | 2.532002937 | 0.14 |
| SAM6964a6d7b967 | 1.176470588 | TC1 | SD/PD | 16.26283368 | dead | -2.557036486 | low | 1.860728672 | -0.71 |
| SAM6cb230f208a8 | 1.039215686 | TC1 | CR/PR | 23.22792608 | alive | -3.78477962 | low | 2.160075409 | 0.08 |
| SAM6cbc10abddb0 | 0.960784314 | TC0 | SD/PD | 5.94661191 | dead | 2.800740977 | high | 2.847851199 | -0.56 |
| SAM6d2ae0c39b96 | NA | TC0 | SD/PD | 22.11088296 | alive | -0.314132203 | high | 2.39436054 | -0.9 |
| SAM6dd7ad1d797d | 3.568627451 | TC2+ | SD/PD | 19.28542094 | dead | 0.992546715 | high | 2.929569391 | 0.9 |
| SAM6f2a102a99df | 2.078431373 | TC2+ | CR/PR | 19.28542094 | alive | 0.634468301 | high | 3.033806344 | -0.36 |
| SAM6ff654a20f98 | 1.039215686 | TC0 | CR/PR | 18.56262834 | alive | -3.49166283 | low | 2.509528682 | 1.04 |
| SAM7114d99032ec | 2.176470588 | TC0 | SD/PD | 10.84188912 | dead | -0.430724617 | high | 2.027582981 | 0.43 |
| SAM716f54e468f4 | 1.568627451 | TC0 | SD/PD | 21.19096509 | alive | 0.419253696 | high | 2.726467329 | 0.64 |
| SAM727c0e92a2a7 | 0.333333333 | TC2+ | CR/PR | 18.72689938 | alive | -0.08807939 | high | 3.194496917 | 0.72 |
| SAM73663ee4a96e | NA | TC0 | CR/PR | 21.25667351 | alive | -3.619149889 | low | 2.805487501 | -0.84 |
| SAM75142fcab9df | 0.274509804 | TC0 | SD/PD | 11.92607803 | dead | 3.239566642 | high | 2.610871144 | -0.32 |
| SAM7538ad9ff524 | 2.392156863 | TC0 | SD/PD | 19.1211499 | alive | -1.736772282 | low | 2.588976353 | -0.98 |
| SAM753d4bb52dbe | NA | TC0 | SD/PD | 11.36755647 | dead | 1.19259024 | high | 2.507661679 | 0.68 |
| SAM75f12d1a55fc | 0.333333333 | TC0 | SD/PD | 14.75154004 | alive | -0.022362315 | high | 2.969854502 | 1 |
| SAM76a431ba6ce1 | 0.215686275 | TC0 | SD/PD | 14.12731006 | dead | 4.940204986 | high | 2.65051409 | 0.67 |
| SAM771445e92421 | 0.215686275 | TC0 | CR/PR | 19.41683778 | alive | 1.782358049 | high | 2.868039549 | 0.92 |
| SAM7829a341b9f3 | 0.745098039 | TC1 | SD/PD | 3.876796715 | dead | -3.907308749 | low | 2.665719242 | -0.93 |
| SAM7893196e0e89 | 0.941176471 | TC0 | SD/PD | 2.661190965 | dead | -0.203847415 | high | 2.337302338 | -0.09 |
| SAM7aa01fc49a80 | 1.039215686 | TC0 | SD/PD | 2.135523614 | dead | 4.214741503 | high | 2.756947222 | 0.63 |
| SAM7b40007f4aa4 | NA | TC0 | CR/PR | 21.65092402 | alive | -8.804383204 | low | 1.736136012 | -0.23 |
| SAM7bff231634e9 | 0.725490196 | TC2+ | SD/PD | 10.25051335 | dead | -6.390895262 | low | 2.964736714 | -0.31 |
| SAM7c67b05aa109 | 0.529411765 | TC1 | SD/PD | 8.246406571 | dead | -1.117631233 | low | 1.802498556 | -0.59 |
| SAM7d2dfba6cd84 | 0.156862745 | TC0 | SD/PD | 12.71457906 | dead | 0.518260447 | high | 2.85091519 | 0.01 |
| SAM7d7c54623618 | 2.098039216 | TC0 | CR/PR | 21.2238193 | alive | -1.532412376 | low | 2.207817098 | 0.16 |
| SAM7f0d9cc7f001 | NA | TC0 | SD/PD | 4.632443532 | dead | 1.018284027 | high | 2.582488864 | 0.42 |
| SAM7fb6987514a4 | NA | TC0 | SD/PD | 22.83367556 | alive | 1.807713445 | high | 3.203254542 | 0.71 |
| SAM7fb7a13c096b | 0.764705882 | TC0 | SD/PD | 1.675564682 | dead | -4.452607679 | low | 2.530026357 | 0.32 |
| SAM80c6183220e6 | 0.215686275 | TC0 | SD/PD | 14.75154004 | alive | -1.075466897 | low | 2.900475938 | -0.18 |
| SAM81b71522417a | 1.078431373 | TC0 | SD/PD | 2.135523614 | dead | -1.049300702 | low | 1.914714912 | -0.29 |
| SAM822b226466a1 | 1.098039216 | TC0 | SD/PD | 17.28131417 | alive | -3.201516119 | low | 2.041763398 | -0.67 |
| SAM8533e5e261d6 | 1.725490196 | TC0 | CR/PR | 12.81314168 | dead | -0.177288031 | high | 2.096946873 | -0.77 |
| SAM85f0a3ac1c45 | 0.274509804 | TC0 | SD/PD | 5.848049281 | dead | 1.719773236 | high | 2.030391766 | -0.38 |
| SAM87a8e18eb45b | 0.31372549 | TC0 | SD/PD | 2.759753593 | dead | -2.355601231 | low | 2.208340435 | -0.77 |
| SAM8884fe446d20 | 6 | TC2+ | CR/PR | 23.16221766 | alive | 5.45424015 | high | 3.238752245 | 0.3 |
| SAM8a1b0e02ee42 | 0.843137255 | TC0 | CR/PR | 20.56673511 | alive | -3.181199117 | low | 2.142588742 | -0.28 |
| SAM8b4b8b0f9e73 | 1 | TC0 | SD/PD | 15.67145791 | alive | 1.094323649 | high | 2.80753095 | 0.64 |
| SAM8e469834acc1 | NA | TC0 | SD/PD | 3.712525667 | dead | 2.493451546 | high | 2.452145939 | 1.53 |
| SAM8e8ef2368dfa | 0.509803922 | TC0 | SD/PD | 4.501026694 | dead | 2.251069036 | high | 2.357107044 | 1.93 |
| SAM8f2275c36e8c | NA | TC0 | SD/PD | 20.73100616 | alive | 1.241023298 | high | 2.190360197 | -0.58 |
| SAM91c47b054ffb | 2.705882353 | TC0 | CR/PR | 17.01848049 | dead | -2.711292202 | low | 2.784087864 | -0.59 |
| SAM9306c5c92444 | 2.235294118 | TC1 | SD/PD | 15.44147844 | alive | -2.558745905 | low | 2.640983499 | -1.23 |
| SAM9410b866974a | NA | TC0 | SD/PD | 6.735112936 | dead | 0.273374891 | high | 2.661806116 | 0.46 |
| SAM943df5cf15df | 0.117647059 | TC0 | SD/PD | 2.135523614 | dead | 3.051628114 | high | 2.462687442 | 0.85 |
| SAM94859b440b1d | 0.215686275 | TC0 | SD/PD | 13.33880903 | dead | -2.480469547 | low | 2.120045371 | -0.29 |
| SAM9539a4f19ebc | 0.431372549 | TC0 | SD/PD | 7.950718686 | dead | 0.411253162 | high | 2.037187257 | 0.16 |
| SAM957378bd907f | 0.196078431 | TC0 | SD/PD | 2.234086242 | dead | 1.429728899 | high | 3.030443971 | 0.93 |
| SAM961d04c42bd9 | 1.725490196 | TC0 | SD/PD | 5.880903491 | dead | 0.716967552 | high | 2.40061133 | -0.23 |
| SAM9725303dce0c | 4.431372549 | TC0 | SD/PD | 8.90349076 | alive | -6.810354866 | low | 2.401116915 | -1.15 |
| SAM978a587b207e | 1.921568627 | TC2+ | SD/PD | 2.102669405 | dead | 0.017448645 | high | 2.723132238 | 1.05 |
| SAM97a00e0929fb | 2.862745098 | TC0 | CR/PR | 12.71457906 | alive | -2.928513433 | low | 2.624765788 | -0.49 |
| SAM99a46b9eec27 | 0.725490196 | TC0 | SD/PD | 3.613963039 | dead | 2.156450023 | high | 2.669634467 | 0.16 |
| SAM9a2cf3c06fb3 | 0.862745098 | TC0 | SD/PD | 5.650924025 | dead | 2.216636302 | high | 2.642667052 | 0.68 |
| SAM9aa6a095a9d6 | 0.647058824 | TC0 | SD/PD | 8.837782341 | dead | 1.177421334 | high | 2.509546181 | -0.04 |
| SAM9cafb905b36a | 0.588235294 | TC2+ | SD/PD | 7.622176591 | dead | 1.793303368 | high | 3.137044982 | 0.21 |
| SAM9d2494119c05 | NA | TC0 | SD/PD | 1.445585216 | dead | 0.172824011 | high | 2.097048509 | -0.4 |
| SAM9daccafc18db | NA | TC0 | SD/PD | 16.45995893 | alive | -1.011713055 | low | 2.442620032 | 1.7 |
| SAM9e11ec6bea80 | NA | TC2+ | SD/PD | 15.40862423 | dead | 3.40196924 | high | 3.091553841 | -0.18 |
| SAM9fb814c22bdb | 0.431372549 | TC0 | SD/PD | 19.1211499 | dead | 5.555680097 | high | 2.904620695 | 0.84 |
| SAMa1871f491b02 | 3.333333333 | TC0 | SD/PD | 1.741273101 | dead | -2.35249442 | low | 2.372729561 | -1.4 |
| SAMa1e62d323e1d | 0.921568627 | TC0 | SD/PD | 10.57905544 | dead | -0.238612263 | high | 2.657928651 | 0.26 |
| SAMa321770ac31c | 1.411764706 | TC0 | SD/PD | 7.063655031 | alive | -2.945718847 | low | 2.68614698 | -0.24 |
| SAMa424c75831b4 | 0.68627451 | TC0 | SD/PD | 3.54825462 | dead | 1.80259939 | high | 2.72991978 | -0.07 |
| SAMa535fcdf18a0 | 0.882352941 | TC0 | CR/PR | 17.28131417 | alive | 1.465579975 | high | 2.361321987 | 0.3 |
| SAMa90d73f8d891 | 0.725490196 | TC0 | SD/PD | 1.018480493 | dead | 0.581568302 | high | 1.94809494 | 0.08 |
| SAMa913c6139ec8 | 1.529411765 | TC0 | SD/PD | 5.125256674 | dead | 1.408404561 | high | 2.833781631 | -0.84 |
| SAMa9ca8536d2b1 | 0.352941176 | TC1 | SD/PD | 21.84804928 | alive | 2.679527653 | high | 2.680092729 | 1.22 |
| SAMaabf4afe4213 | 2 | TC0 | SD/PD | 2.628336756 | dead | -4.129869359 | low | 2.400729705 | 0.29 |
| SAMaaf505c36f93 | 1.725490196 | TC0 | CR/PR | 20.56673511 | alive | 0.265926316 | high | 2.363741937 | -0.03 |
| SAMab8052a03398 | 0.039215686 | TC0 | SD/PD | 13.30595483 | dead | 2.011722404 | high | 2.880186511 | 1.03 |
| SAMabc151b01ea3 | NA | TC0 | SD/PD | 0.558521561 | dead | 1.58083161 | high | 2.226132518 | -0.3 |
| SAMad83c9c53537 | 0.745098039 | TC0 | SD/PD | 14.98151951 | alive | -0.176231322 | high | 2.391054433 | 1 |
| SAMae02629a97f7 | NA | TC0 | SD/PD | 3.252566735 | dead | -3.925807924 | low | 2.07352115 | 0.16 |
| SAMae1690469964 | NA | TC0 | SD/PD | 21.15811088 | dead | -1.118256489 | low | 2.497899723 | -1.53 |
| SAMae4da274eded | NA | TC0 | SD/PD | 4.993839836 | dead | -4.448058999 | low | 2.640056725 | -0.69 |
| SAMaec7380f9ab0 | NA | TC0 | CR/PR | 20.862423 | alive | -1.627198668 | low | 2.305041796 | -0.38 |
| SAMaf42c1541269 | 2.196078431 | TC0 | SD/PD | 5.519507187 | alive | -0.353888465 | high | 3.100742003 | 0.28 |
| SAMb0a83e5fbde9 | 0.352941176 | TC0 | SD/PD | 23.32648871 | alive | -0.28429131 | high | 2.344834613 | -0.48 |
| SAMb0d11db9aa79 | 0.843137255 | TC0 | SD/PD | 10.54620123 | dead | 2.106445314 | high | 3.06631302 | 0.41 |
| SAMb15ac6e4c4ef | 1.509803922 | TC0 | CR/PR | 20.00821355 | alive | -2.506003068 | low | 2.833670402 | -1.55 |
| SAMb15ad09d6e24 | NA | TC2+ | SD/PD | 3.876796715 | dead | 2.507749204 | high | 3.194359524 | 0.53 |
| SAMb2e4a082541a | NA | TC0 | SD/PD | 4.369609856 | dead | -1.155821068 | low | 2.950341277 | -2.05 |
| SAMb2f1d0e54ece | 0.862745098 | TC2+ | CR/PR | 11.40041068 | dead | -0.190268664 | high | 2.814618898 | 1.52 |
| SAMb3c02294aba7 | NA | TC1 | CR/PR | 22.40657084 | alive | 1.024860456 | high | 2.059552885 | -0.59 |
| SAMb419a8fcbfcd | NA | TC0 | CR/PR | 22.73511294 | alive | 1.079254944 | high | 2.125028132 | -0.03 |
| SAMb470eb8f04be | NA | TC0 | CR/PR | 20.07392197 | alive | 1.490739914 | high | 2.548337886 | -0.25 |
| SAMb4c7a001537d | NA | TC1 | SD/PD | 3.186858316 | dead | 0.333251484 | high | 2.609503655 | -1.03 |
| SAMb8070b7937e7 | 0.333333333 | TC0 | SD/PD | 21.02669405 | dead | 2.250808999 | high | 2.350759765 | -0.51 |
| SAMb8101c538753 | 3.764705882 | TC2+ | SD/PD | 13.40451745 | dead | 0.891260631 | high | 2.986648981 | 0.19 |
| SAMb963dda93cfd | 0.882352941 | TC2+ | SD/PD | 2.759753593 | dead | -0.464559135 | high | 2.566846592 | 0.78 |
| SAMba1a34b5a060 | 0.901960784 | TC0 | SD/PD | 12.84599589 | dead | -0.857644507 | high | 2.663635519 | 0.78 |
| SAMba7176afe070 | NA | TC0 | SD/PD | 3.449691992 | alive | 1.196572506 | high | 2.024655944 | -0.98 |
| SAMbc8dc3a7b54e | 0.745098039 | TC0 | SD/PD | 14.75154004 | dead | -6.099555831 | low | 1.814317708 | -0.83 |
| SAMbcb07ba81cee | NA | TC0 | SD/PD | 2.529774127 | dead | 1.290498769 | high | 2.668201278 | -0.2 |
| SAMbcbc7957c264 | NA | TC0 | SD/PD | 4.1724846 | dead | 0.967519151 | high | 2.21349018 | -0.48 |
| SAMbda79f955628 | 1.254901961 | TC0 | SD/PD | 20.63244353 | alive | -0.86080715 | low | 3.265072925 | 0.61 |
| SAMbe83eae4026e | 0.784313725 | TC0 | SD/PD | 2.726899384 | dead | 0.917780396 | high | 2.523516641 | -0.36 |
| SAMbf1a3ae828e6 | 3.745098039 | TC0 | CR/PR | 20.73100616 | alive | 2.407040931 | high | 2.624091049 | 1.03 |
| SAMbfdffb97c446 | 1.450980392 | TC2+ | SD/PD | 6.702258727 | dead | -0.11452567 | high | 2.738034004 | 0.67 |
| SAMc0d625a50eb8 | 0.294117647 | TC0 | SD/PD | 13.27310062 | dead | 3.558344478 | high | 2.379742153 | 2.34 |
| SAMc0da5d48686d | 1.176470588 | TC2+ | SD/PD | 9.49486653 | dead | 3.95324772 | high | 2.384424566 | 0.07 |
| SAMc0ef41aa6c8b | 0.960784314 | TC0 | SD/PD | 9.560574949 | dead | -2.779560276 | low | 1.713817047 | -0.71 |
| SAMc1251c7bfee2 | 1.098039216 | TC0 | CR/PR | 21.61806982 | alive | 0.110071533 | high | 2.610768748 | -0.41 |
| SAMc1b27bc16435 | 1.411764706 | TC0 | CR/PR | 24.18069815 | alive | -1.174189521 | low | 2.612585914 | -0.65 |
| SAMc2a1820d4e6b | NA | TC0 | CR/PR | 20.73100616 | alive | -0.093122384 | high | 2.887389357 | 0.71 |
| SAMc57eadb2d82b | 0.196078431 | TC0 | SD/PD | 8.016427105 | dead | -4.512772644 | low | 1.740038091 | -0.61 |
| SAMc692536a795a | NA | TC0 | SD/PD | 5.650924025 | dead | 0.426299358 | high | 2.699600194 | 1.3 |
| SAMc6eff056c89a | 0.450980392 | TC0 | SD/PD | 1.80698152 | dead | -0.703266327 | high | 2.597258377 | -0.56 |
| SAMc919aebc7fdd | 0.607843137 | TC0 | CR/PR | 20.73100616 | alive | 2.81254562 | high | 2.950462816 | -0.7 |
| SAMcabb6d58ff55 | 1.352941176 | TC2+ | SD/PD | 20.27104723 | alive | -3.866993144 | low | 2.926972079 | 0.58 |
| SAMcb132b0cdd2c | 0.843137255 | TC2+ | SD/PD | 2.529774127 | dead | -3.54802674 | low | 2.844574355 | -0.89 |
| SAMcc4675f394a1 | NA | TC0 | SD/PD | 3.121149897 | dead | 1.554721546 | high | 2.251572794 | -0.06 |
| SAMcc7a42d87e9c | 4.960784314 | TC0 | CR/PR | 17.21560575 | alive | -3.280812426 | low | 2.754358804 | -0.33 |
| SAMce39dd79b441 | 0.843137255 | TC0 | SD/PD | 6.275154004 | dead | -3.600536263 | low | 2.560355262 | 0.03 |
| SAMcf018fee2acd | NA | TC0 | SD/PD | 16.22997947 | dead | 2.048290365 | high | 2.440335946 | -1.13 |
| SAMd027124354ce | 0.764705882 | TC0 | CR/PR | 23.52361396 | alive | 0.721145804 | high | 2.279407137 | 1.9 |
| SAMd135d5867fe3 | 0.607843137 | TC2+ | SD/PD | 3.613963039 | dead | 0.699334693 | high | 2.847297357 | 0.73 |
| SAMd1bd63734394 | NA | TC0 | SD/PD | 1.938398357 | dead | -2.353903369 | low | 2.786403694 | -1.05 |
| SAMd215b503f99a | NA | TC0 | SD/PD | 1.708418891 | alive | 0.361296076 | high | 2.406028057 | 2.63 |
| SAMd2492b2a31bb | 0.490196078 | TC2+ | SD/PD | 7.852156057 | dead | 4.006163321 | high | 2.677679868 | 2.68 |
| SAMd35318127278 | 10.01960784 | TC1 | CR/PR | 21.58521561 | alive | -2.458292308 | low | 2.753099419 | -0.99 |
| SAMd3601288319e | 0.784313725 | TC0 | CR/PR | 23.12936345 | alive | 2.326594369 | high | 2.960693081 | -0.22 |
| SAMd3bd67996035 | 1.568627451 | TC0 | CR/PR | 20.82956879 | alive | 0.253326762 | high | 2.877970543 | -1.42 |
| SAMd43f8933066b | 1.882352941 | TC0 | CR/PR | 20.00821355 | alive | 2.017155482 | high | 2.953138236 | 0.39 |
| SAMd4c0837b0997 | 0.529411765 | TC1 | SD/PD | 5.388090349 | dead | 3.063623687 | high | 2.497485518 | -0.26 |
| SAMd636e3461955 | 0.196078431 | TC0 | SD/PD | 10.48049281 | dead | 4.159928781 | high | 2.660897432 | 1.7 |
| SAMd697ba701077 | NA | TC0 | SD/PD | 21.15811088 | dead | -1.272365396 | low | 2.664366949 | -0.74 |
| SAMd7d57ee3a863 | NA | TC0 | CR/PR | 20.27104723 | alive | 2.251465986 | high | 3.182869725 | 0.3 |
| SAMd86389d0d768 | 1.411764706 | TC0 | SD/PD | 2.201232033 | dead | 0.439493986 | high | 2.031021859 | -0.85 |
| SAMd98bac0a070f | 0.725490196 | TC0 | SD/PD | 2.069815195 | dead | 2.838303173 | high | 2.263660703 | 0.18 |
| SAMda4d892fddc8 | 0.274509804 | TC0 | SD/PD | 14.12731006 | alive | -0.829707265 | high | 2.650347689 | 0.68 |
| SAMdab9ca8fb5de | 2.921568627 | TC0 | SD/PD | 0.197125257 | alive | 0.143146474 | high | 2.443771063 | 0.77 |
| SAMdad5c29dc105 | 0.666666667 | TC0 | SD/PD | 2.431211499 | dead | -1.739856216 | low | 2.437633074 | 0.39 |
| SAMdb3f50c9129c | 0.509803922 | TC0 | SD/PD | 15.80287474 | dead | -1.0175089 | low | 2.261696287 | 0.43 |
| SAMdcae54fcd7fa | 1.431372549 | TC0 | SD/PD | 6.242299795 | dead | -0.803880694 | high | 2.507035869 | -0.05 |
| SAMdee1011782cd | NA | TC0 | SD/PD | 3.909650924 | dead | 0.426407373 | high | 2.921864369 | 0.82 |
| SAMdf3e42c8672a | 3.235294118 | TC2+ | SD/PD | 7.326488706 | dead | 0.148238279 | high | 2.810013855 | 0.31 |
| SAMe07c4560772d | 0.549019608 | TC0 | SD/PD | 2.102669405 | alive | -4.430417936 | low | 1.715334359 | -0.69 |
| SAMe0c49ea0df5d | NA | TC0 | SD/PD | 4.13963039 | dead | -1.39184297 | low | 2.587942481 | 1.64 |
| SAMe1eb5d988760 | 2.274509804 | TC0 | CR/PR | 21.05954825 | alive | 0.913709318 | high | 2.91475382 | 0.52 |
| SAMe3210d3632b4 | 0.862745098 | TC2+ | SD/PD | 2.168377823 | dead | 4.633868281 | high | 2.881884543 | 0.64 |
| SAMe3d4266775a9 | NA | TC0 | SD/PD | 13.99589322 | alive | -5.807078611 | low | 2.400330414 | 0.19 |
| SAMe41b1e773582 | 0.470588235 | TC0 | SD/PD | 0.854209446 | dead | -0.587081241 | high | 2.626956116 | -0.15 |
| SAMe50d15fde368 | 2.176470588 | TC0 | CR/PR | 18.89117043 | alive | -2.899448181 | low | 1.991586866 | -0.69 |
| SAMe56c96c51190 | 0.098039216 | TC2+ | SD/PD | 9.757700205 | dead | 1.372350396 | high | 2.926707279 | 0.49 |
| SAMe5bc41772bc9 | 0.274509804 | TC0 | SD/PD | 6.110882957 | dead | 1.87130774 | high | 2.246138882 | 0.35 |
| SAMe712352fb82a | NA | TC0 | CR/PR | 22.11088296 | alive | 0.130783525 | high | 2.332542578 | -0.96 |
| SAMe7bcab05402e | NA | TC0 | SD/PD | 7.917864476 | dead | 2.410525537 | high | 2.303753254 | 1.95 |
| SAMe7bf6c015192 | 1.37254902 | TC0 | SD/PD | 3.121149897 | dead | 1.399245166 | high | 2.907616669 | -0.72 |
| SAMe7e4f7c076a7 | 0.196078431 | TC2+ | SD/PD | 1.971252567 | dead | 1.648141478 | high | 3.135576416 | 0.45 |
| SAMe9475f77504b | NA | TC0 | SD/PD | 18.66119097 | alive | 4.05368963 | high | 2.664451146 | 1.49 |
| SAMe94c30c30616 | 1.019607843 | TC0 | SD/PD | 15.37577002 | dead | -0.807959599 | high | 2.648811266 | 0.86 |
| SAMe97af0feefdf | 0.705882353 | TC0 | SD/PD | 11.10472279 | dead | -1.451769041 | low | 2.361703909 | 0.68 |
| SAMe9ae8beb82fa | 1.333333333 | TC0 | SD/PD | 5.486652977 | dead | 1.652624476 | high | 2.493084601 | 0.92 |
| SAMeaa477a5384b | 2.764705882 | TC0 | CR/PR | 21.58521561 | alive | -2.574185538 | low | 3.020015238 | 0.4 |
| SAMeb29625f76a5 | 0.058823529 | TC0 | SD/PD | 3.121149897 | dead | 1.034920516 | high | 2.52516209 | -0.5 |
| SAMeb587a68006b | NA | TC0 | SD/PD | 20.69815195 | alive | 3.304172985 | high | 2.474465751 | 1.99 |
| SAMee3844cc0b9f | NA | TC0 | SD/PD | 3.219712526 | dead | 1.767948127 | high | 2.911397156 | 1.82 |
| SAMef0e3d2415fd | 0.039215686 | TC2+ | SD/PD | 10.11909651 | dead | -0.002509009 | high | 3.423623915 | 1.51 |
| SAMeff2ce356ccb | NA | TC0 | SD/PD | 8.082135524 | dead | -0.383481429 | high | 2.272085859 | -0.81 |
| SAMf20b827dca51 | 1.098039216 | TC0 | SD/PD | 4.369609856 | dead | -0.607609219 | high | 3.062191967 | 1.03 |
| SAMf275eb859a39 | NA | TC0 | CR/PR | 24.47638604 | alive | -1.493965096 | low | 2.59825892 | -0.98 |
| SAMf28c01545593 | NA | TC0 | SD/PD | 6.406570842 | dead | -0.612806291 | high | 1.963455639 | -0.92 |
| SAMf2aae1443f67 | 0.803921569 | TC0 | SD/PD | 10.34907598 | dead | -0.363852013 | high | 2.232237905 | -0.47 |
| SAMf3a9bce50099 | NA | TC0 | SD/PD | 2.102669405 | alive | 0.135891028 | high | 2.687694763 | 1.03 |
| SAMf82bbdc267c8 | 0.529411765 | TC0 | SD/PD | 3.121149897 | dead | -0.488881563 | high | 1.870262675 | -0.09 |
| SAMfb7aec7cb0e2 | 5.921568627 | TC2+ | CR/PR | 22.53798768 | alive | 0.400969836 | high | 3.026827309 | -0.97 |
| SAMfd947610629d | NA | TC0 | SD/PD | 9.034907598 | dead | 0.965481791 | high | 2.252197456 | 2.28 |
| SAMfed609955db9 | 0.352941176 | TC2+ | SD/PD | 20.56673511 | alive | 2.797554396 | high | 2.196787929 | 0.96 |
| SAMffa5c7cad0e5 | NA | TC0 | CR/PR | 15.54004107 | dead | 3.083217789 | high | 2.544889915 | 2.04 |

***** TC0, TC1, and TC2+ represents <1%, ≥1% but <5%, ≥5% of tumor cells were PD-L1 positive, respectively.
