# Supplementary material for: m6A-Regulator Expression Signatures Identify a Subset of Follicular Lymphoma Harboring an Exhausted Tumor Microenvironment
Source: Front Immunol. 2022 Jun 6;13:922471. doi: 10.3389/fimmu.2022.922471 (PMC9207509; doi:10.3389/fimmu.2022.922471)
Supplement: Supplementary Figure 1 — Flowchart of the study design. FL, follicular lymphoma; DLBCL, diffuse large B cell lymphoma; OS, overall survival; TMUCH, Tianjin Medical University Cancer Institute and Hospital. [file DataSheet_2.pdf]

Figure S1

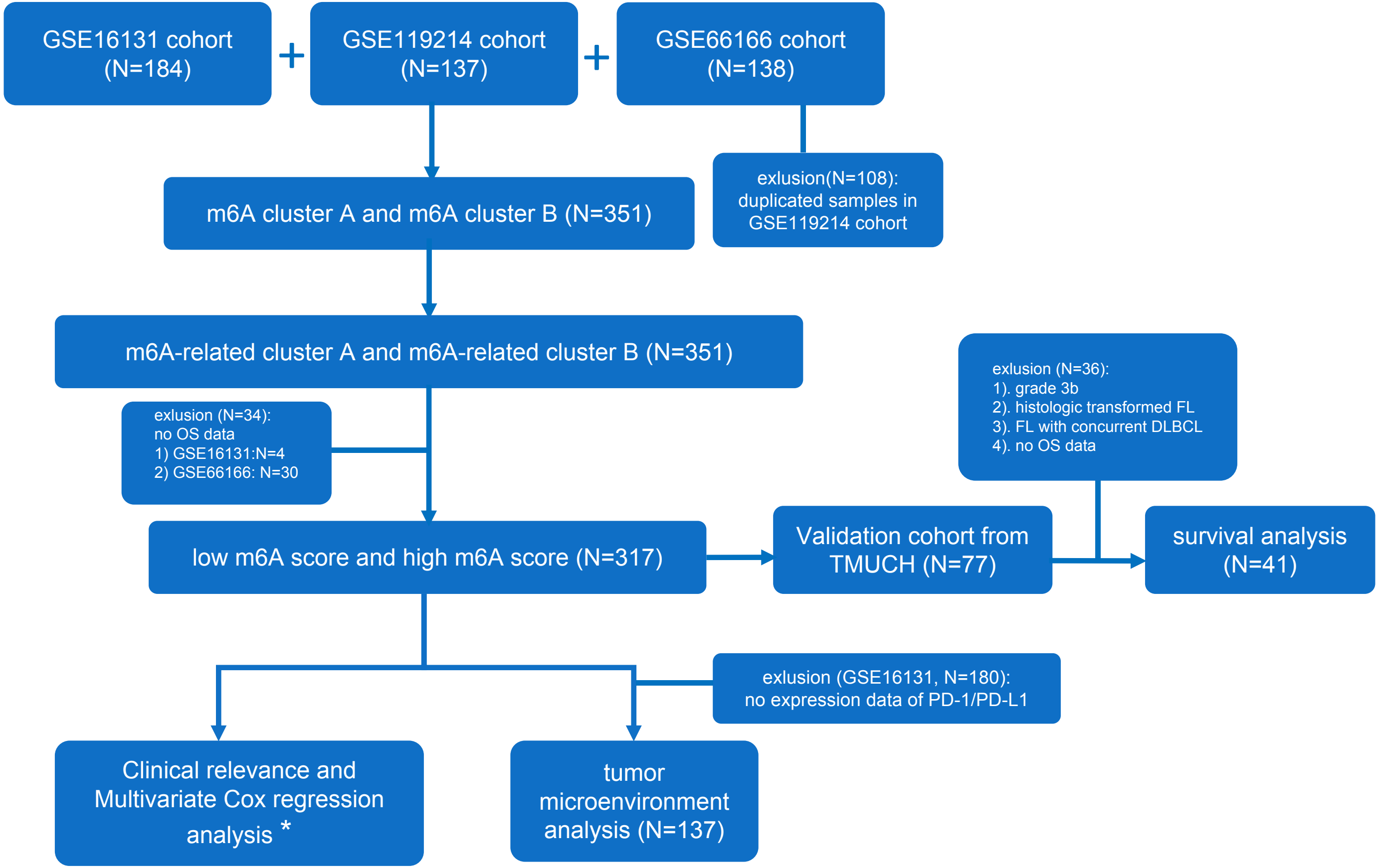


* including any samples as long as corresponding clinical parameters were available

Figure S2

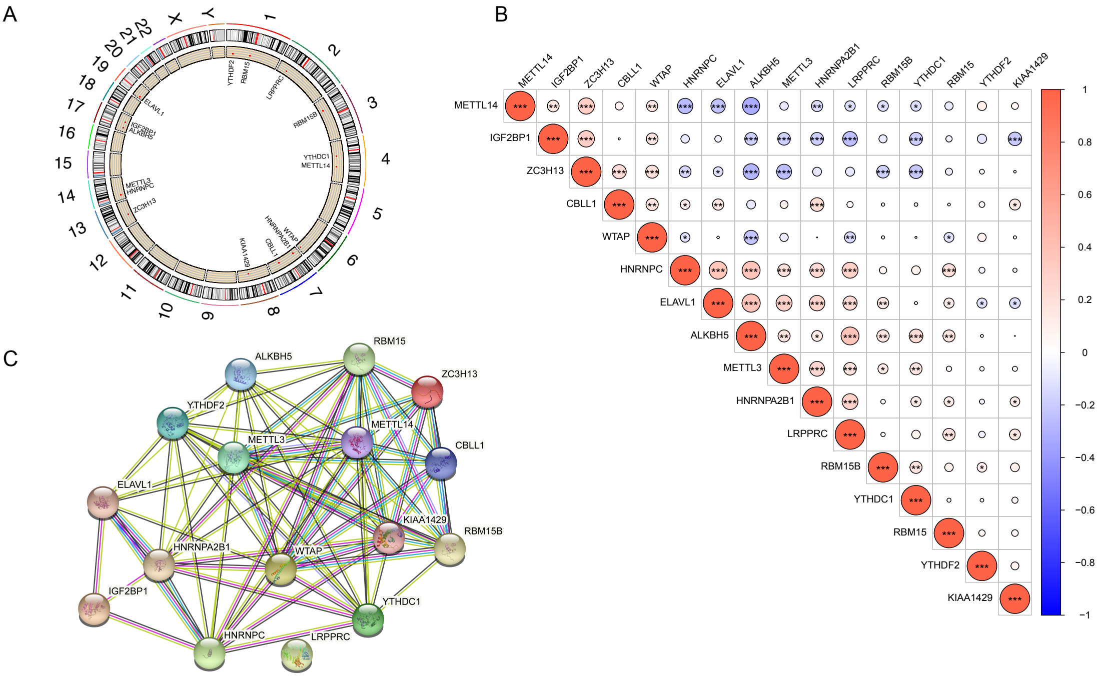

A
1
2
3
4
5
6
7
8
9
10
11
12
13
14
15
16
17
18
19
20
21
22
X
Y
YTHDF2
RBM15
LRPPRC
RBM15B
YTHDC1
METTL14
ELAVL1
IGF2BP1
ALKBH5
METTL3
HNRNPC
ZC3H13
HNRNPA2B1
WTAP
CBLL1
KIAA1429
B
METTL14
IGF2BP1
ZC3H13
CBLL1
WTAP
HNRNPC
ELAVL1
ALKBH5
METTL3
HNRNPA2B1
LRPPRC
RBM15B
YTHDC1
RBM15
YTHDF2
KIAA1429
1
0.8
0.6
0.4
0.2
0
−0.2
−0.4
−0.6
−0.8
−1
C
RBM15
ALKBH5
ZC3H13
YTHDF2
METTL14
METTL3
CBLL1
ELAVL1
KIAA1429
RBM15B
HNRNPA2B1
WTAP
IGF2BP1
YTHDC1
HNRNPC
LRPPRC

# Figure S3

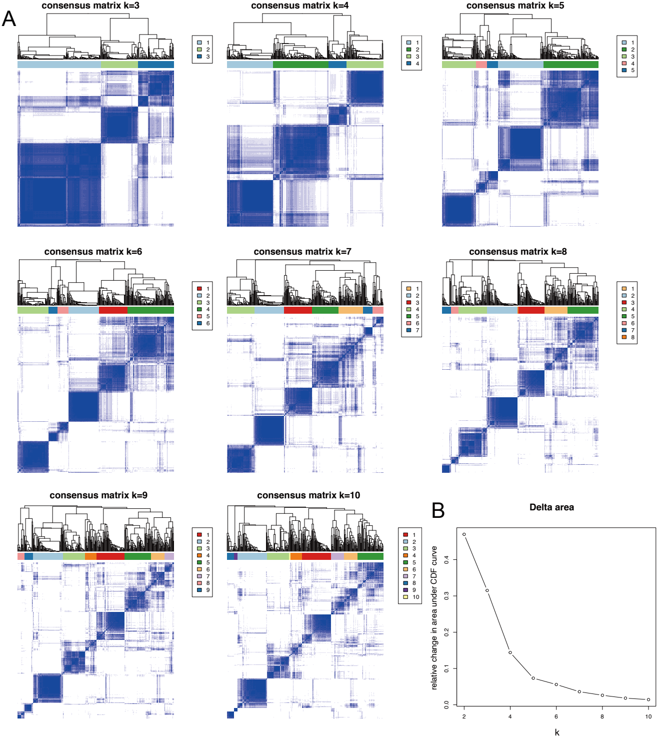
A
consensus matrix k=3
consensus matrix k=4
consensus matrix k=5
consensus matrix k=6
consensus matrix k=7
consensus matrix k=8
consensus matrix k=9
consensus matrix k=10
1 2 3 4 5 6 7 8 9 10
B
Delta area
relative change in area under CDF curve
0.0 0.1 0.2 0.3 0.4
2 4 6 8 10
k

Figure S4

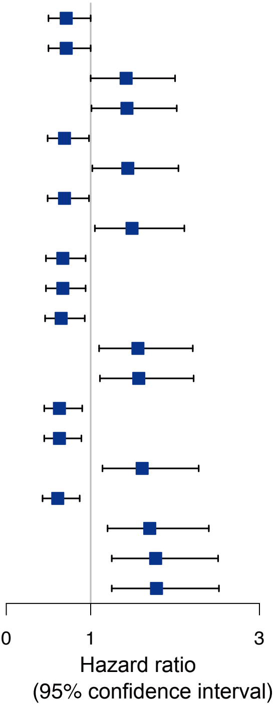


| | p value | Hazard ratio |
|---|---|---|
| TMEM203 | 0.049 | 0.71 (0.5–1) |
| DDX54 | 0.048 | 0.71 (0.5–1) |
| MAPK8 | 0.048 | 1.42 (1–2) |
| ZNF436 | 0.043 | 1.43 (1.01–2.02) |
| MIF4GD | 0.038 | 0.69 (0.49–0.98) |
| RNF219 | 0.038 | 1.44 (1.02–2.04) |
| NDUFAF2 | 0.036 | 0.69 (0.49–0.98) |
| SCFD1 | 0.024 | 1.49 (1.05–2.11) |
| SNX2 | 0.023 | 0.67 (0.47–0.94) |
| MRPL14 | 0.02 | 0.67 (0.47–0.94) |
| PCNT | 0.018 | 0.65 (0.46–0.93) |
| MCL1 | 0.012 | 1.56 (1.1–2.21) |
| ZNF830 | 0.011 | 1.57 (1.11–2.22) |
| MCM6 | 0.01 | 0.63 (0.45–0.9) |
| ANKRD16 | 0.009 | 0.63 (0.45–0.89) |
| ARAP1 | 0.007 | 1.61 (1.14–2.28) |
| L2HGDH | 0.006 | 0.61 (0.43–0.87) |
| SPIN1 | 0.003 | 1.7 (1.2–2.4) |
| CAB39 | 0.001 | 1.77 (1.25–2.51) |
| DCTN5 | 0.001 | 1.78 (1.25–2.52) |

Figure S5

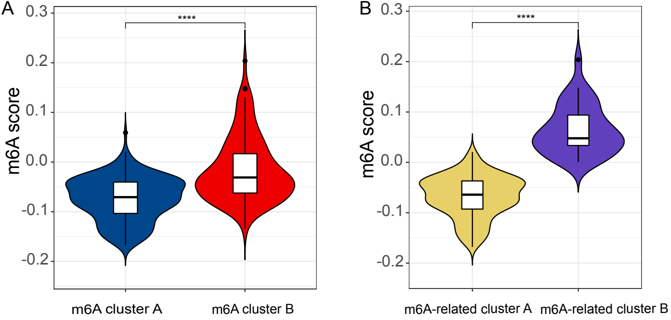
A
B
****
m6A score
0.3
0.2
0.1
0.0
-0.1
-0.2
m6A cluster A
m6A cluster B
m6A-related cluster A
m6A-related cluster B

Figure S6

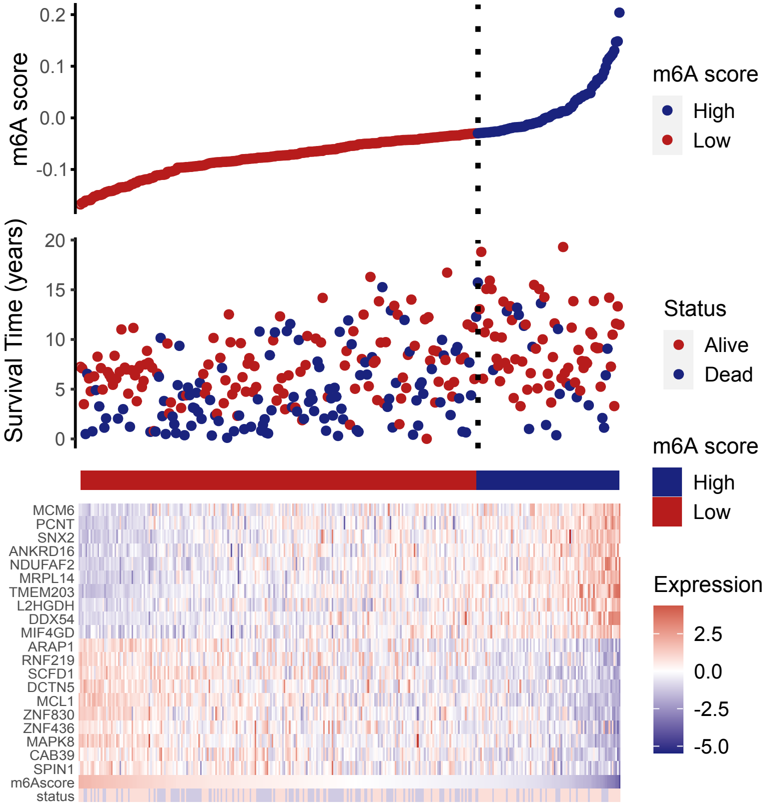

m6A score
0.2
0.1
0.0
-0.1
m6A score
High
Low
Survival Time (years)
20
15
10
5
0
Status
Alive
Dead
m6A score
High
Low
MCM6
PCNT
SNX2
ANKRD16
NDUFAF2
MRPL14
TMEM203
L2HGDH
DDX54
MIF4GD
ARAP1
RNF219
SCFD1
DCTN5
MCL1
ZNF830
ZNF436
MAPK8
CAB39
SPIN1
m6Ascore
status
Expression
2.5
0.0
-2.5
-5.0

Figure S7

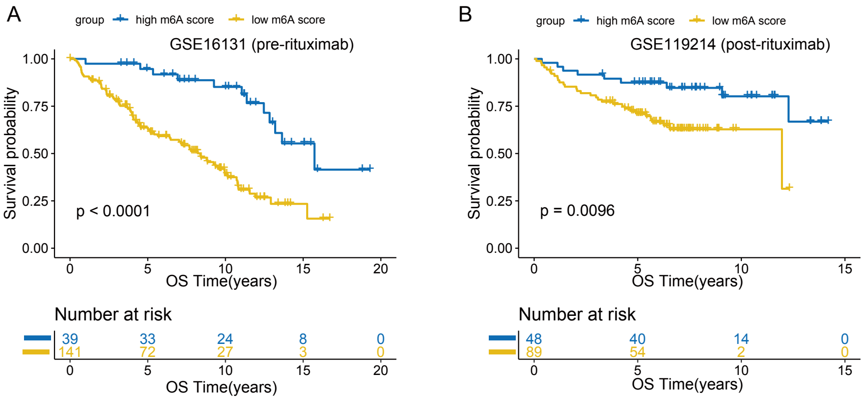
A
group
high m6A score
low m6A score
GSE16131 (pre-rituximab)
Survival probability
1.00
0.75
0.50
0.25
0.00
p < 0.0001
0
5
10
15
20
OS Time(years)
Number at risk
39 33 24 8 0
141 72 27 3 0
OS Time(years)
B
group
high m6A score
low m6A score
GSE119214 (post-rituximab)
Survival probability
p = 0.0096
0
5
10
15
OS Time(years)
Number at risk
48 40 14 0
89 54 2 0
OS Time(years)
